# Physiologically Based Pharmacokinetic Modeling of Inhaled Polymyxin B: From Rabbit Optimization to Human Predictions

**DOI:** 10.1007/s11095-026-04194-1

**Published:** 2026-09-17

**Authors:** Ramya Mahadevan, Shekhar Yeshwante, Rajnikant Sharma, Quentin Valle, ChunFu Cheng, Rani S. Sellers, Maria Soledad Ramirez, Jian Li, Qi Tony Zhou, David Z. D’Argenio, Gauri G. Rao

**Affiliations:** 1 Titus Family Department of Clinical Practice, USC Alfred E. Mann School of Pharmacy and Pharmaceutical Sciences, University of Southern California, Los Angeles, CA USA; 2 Center for Quantitative Drug and Disease Modeling, USC Alfred E. Mann School of Pharmacy and Pharmaceutical Sciences, University of Southern California, Los Angeles, CA USA; 3 Division of Pharmacotherapy and Experimental Therapeutics, UNC Eshelman School of Pharmacy, University of North Carolina at Chapel Hill, Chapel Hill, NC USA; 4 Lineberger Comprehensive Cancer Center, University of North Carolina at Chapel Hill School of Medicine, Chapel Hill, NC USA; 5 Center for Applied Biotechnology Studies, Department of Biological Science, College of Natural Sciences and Mathematics, California State University Fullerton, Fullerton, CA USA; 6 Biomedicine Discovery Institute, Infection Program, Department of Microbiology, Monash University, Melbourne, VIC 3800 Australia; 7 Department of Industrial and Molecular Pharmaceutics, College of Pharmacy, Purdue University, West Lafayette, IN USA; 8 Alfred E. Mann Department of Biomedical Engineering, University of Southern California, Los Angeles, CA USA

**Keywords:** inhalation, PBPK, polymyxin B, rabbit, translational

## Abstract

**Objectives:**

Despite the potential of aerosolized polymyxin B (PMB) to enhance local lung exposure while minimizing nephrotoxicity in multidrug-resistant pneumonia, clinical adoption remains limited by insufficient inhaled pharmacokinetic (PK) data. This study developed a physiologically based pharmacokinetic (PBPK) modeling framework to translate preclinical PK data from rabbits to humans and coupled it with a mechanism-based pharmacodynamic model to define optimal inhaled PMB regimens.

**Methods:**

A whole-body PBPK model was developed using plasma and tissue concentration data following subcutaneous and intratracheal PMB administration (2 mg/kg) in rabbits, extrapolated to humans using allometric scaling, and validated against clinical plasma and epithelial lining fluid (ELF) data. Monte Carlo simulations evaluated the probability of PK/PD target attainment (PTA) and probability of toxicity attainment (PToXA).

**Results:**

Intratracheal administration achieved ~ 10.4-fold higher lung exposure relative to subcutaneous dosing while reducing kidney exposure by 27.7%. A plasma exposure threshold of 92.9 mg·h/L and a kidney tissue exposure threshold of 157 mg·h/L were identified as indicators of higher risk of AKI. Inhaled PMB monotherapy achieved favorable ELF PTA with 0% PToXA and predicted substantial bacterial load reduction (≥ 4 log_10_ CFU/mL at 24 h), whereas IV monotherapy failed to achieve ELF PTA, with several regimens exceeding 40% PToXA and minimal bactericidal activity.

**Conclusion:**

Inhaled PMB provides superior site-specific exposure with minimal predicted nephrotoxicity. Our PBPK-mechanism-based modeling framework suggests that nebulized PMB rapidly clears bacteria while avoiding dose-limiting nephrotoxicity associated with intravenous therapy. These findings support advancing aerosolized PMB toward clinical implementation as a precision-dosing strategy for MDR pulmonary infections.

**Supplementary Information:**

The online version contains supplementary material available at https://doi.org/10.1007/s11095-026-04194-1.

## Introduction

Hospital-acquired pneumonia (HAP) and ventilator-associated pneumonia (VAP) caused by carbapenemase- producing Gram-negative bacteria has high mortality rates and represents a growing therapeutic challenge [1]. Resistance to current therapies such as β-lactam/β-lactamase inhibitors, driven by carbapenemase gene alterations, efflux pumps overexpression, and porin mutations, has limited effective management of these infections [2]. Polymyxins remain among the most widely used antibiotics against carbapenem-resistant Gram-negative bacteria (CR-GNB) in low- and middle-income countries [3], yet poor pulmonary penetration of intravenously (IV) administered polymyxins constrains their efficacy at the pulmonary site of infection.

International consensus guidelines recommend aerosolized formulations as an adjunct to IV therapy for nosocomial pneumonia with multidrug resistant Gram-negative pathogens [4]. However, the majority of preclinical and clinical pharmacokinetic/pharmacodynamic (PK/PD) data on nebulized polymyxins are limited to colistin [5]. Aerosolized colistimethate sodium (CMS), the inactive colistin prodrug, is well established in cystic fibrosis and pneumonia [6, 7]. Unlike CMS which requires *in vivo* hydrolysis to form the active compound colistin, and is associated with higher nephrotoxicity risk and analytical complexity of quantifying both CMS and colistin [8], polymyxin B (PMB) is administered in its active form, is well tolerated in the lungs and requires quantification of a single analyte [9, 10], making it an attractive candidate for inhaled delivery.

Despite these advantages, plasma and epithelial lining fluid (ELF) PK data following inhaled PMB remain scarce, limiting rational dosing strategies. Direct evaluation in humans is particularly challenging, as ELF sampling is invasive, time-consuming, and resource intensive. Physiologically-based pharmacokinetic (PBPK) modeling addresses these limitations by enabling predictions of regional pulmonary drug distribution, including ELF exposure [11, 12]. Furthermore, PBPK models informed by relevant animal data provide a quantitative framework for interspecies scaling to predict human PMB PK. Rabbit models of HAP are ideally suited for PK/PD assessment of aerosolized PMB. Compared with rodents, rabbits are phylogenetically closer to humans, exhibit similar sensitivity to lipopolysaccharide-mediated inflammatory responses, and recapitulate key clinical features of HAP, including severe hypoxemia, neutropenia, hyperlactatemia, and hyperkalemia, while remaining sufficiently large for serial sampling of blood and pulmonary matrices and more economical than pigs or non-human primates [13].

To advance the clinical translation of inhaled PMB, we: (1) characterized PMB PK following intratracheal (IT) administration in rabbits and developed a PBPK model; (2) performed interspecies scaling to predict human exposure and validated against clinical PK data; (3) identified optimal inhalation dosing regimens by evaluating the probability of achieving antibacterial PK/PD targets while minimizing toxicity, using lung and kidney toxicity thresholds derived from population PK/Toxicodynamic (TD) model and published *in vitro* studies [14]; (4) developed a mechanism-based PK/PD model (MBM) using *in vitro* static concentration time-kill (SCTK)data to characterize PMB efficacy against two *Acinetobacter baumannii* strain; and (5) performed simulation-based evaluations comparing inhaled PMB monotherapy with IV monotherapy and combined inhaled-IV regimens, using PBPK-simulated site-specific ELF concentrations as the driver of antibacterial effect.

## Methods

### Polymyxin B PK Following Intratracheal and Subcutaneous Administration in Rabbits

All procedures were approved by the Institutional Animal Care and Use Committee of the University of North Carolina (UNC;protocol #20–021.0). Male and female New Zealand White rabbits (4–6-months old; body weight: 3.31 ± 0.31 kg) with indwelling jugular vein catheters (JVC) were used for PK assessment.

#### Intratracheal Administration

Rabbits (n = 3) were anesthetized via subcutaneous (SC) administration of ketamine (15 mg/kg) and dexmedetomidine (0.125 mg/kg). Following confirmed endotracheal intubation (***Supplementary Methods***) PMB dissolved in sodium chloride for injection (Hospira, Lake Forest, IL), was administered as a single 2 mg/kg intratracheal (IT) dose using a custom-made hand-held MicroSprayer Aerosolizer designed for rabbits (Shanghai Yuyan Instruments, Shanghai, China). The dose was chosen based on a previous study demonstrating that a 2 mg/kg/day IV dose in rabbits achieved human-equivalent exposure [15]. Anesthesia was reversed with SC atipamezole (1.25 mg/kg), followed by endotracheal tube removal. Immunofluorescence (IF) analysis using a polymyxin B-specific monoclonal antibody (anti-PMB mouse IgM antibody; Invitrogen) was used to visualize PMB distribution in kidney tissues over the 6-h study period after IT administration [16]. Tissue samples were stained with mouse anti-PMB antibody (1:500) to localize PMB, and goat anti-mouse IgM conjugated with Alexa Fluor 647 (1:250) was used to visualize PMB distribution.

#### Subcutaneous Administration

Non-anesthetized rabbits (n = 3) received a single SC dose of PMB (2 mg/kg) dissolved in sodium chloride.

#### Sample Collection

Blood samples (~ 1.5 mL) were collected from both administration groups via jugular vein vascular access port at 0.25, 0.5, 1, 1.5, 2, 4, and 6 h post-dose. Terminal tissue samples were collected post-euthanasia. PMB concentrations in plasma, tissues and ELF were quantified as described in the ***Supplementary Methods***.

#### PBPK Model Development

As rabbits are a USDA-protected species, this study was designed in adherence to the 3Rs principles of replacement, reduction, and refinement. Consequently, tissue samples were collected at only three time points (0.5, 2, and 6 h post-dose; n = 3–4 per time point), which limited the characterization of the full tissue disposition profile of PMB from observed data alone. Therefore, a whole-body PBPK model was developed to characterize PMB disposition in plasma and tissues following SC and IT administration [17, 18].

**Fig. 1:**
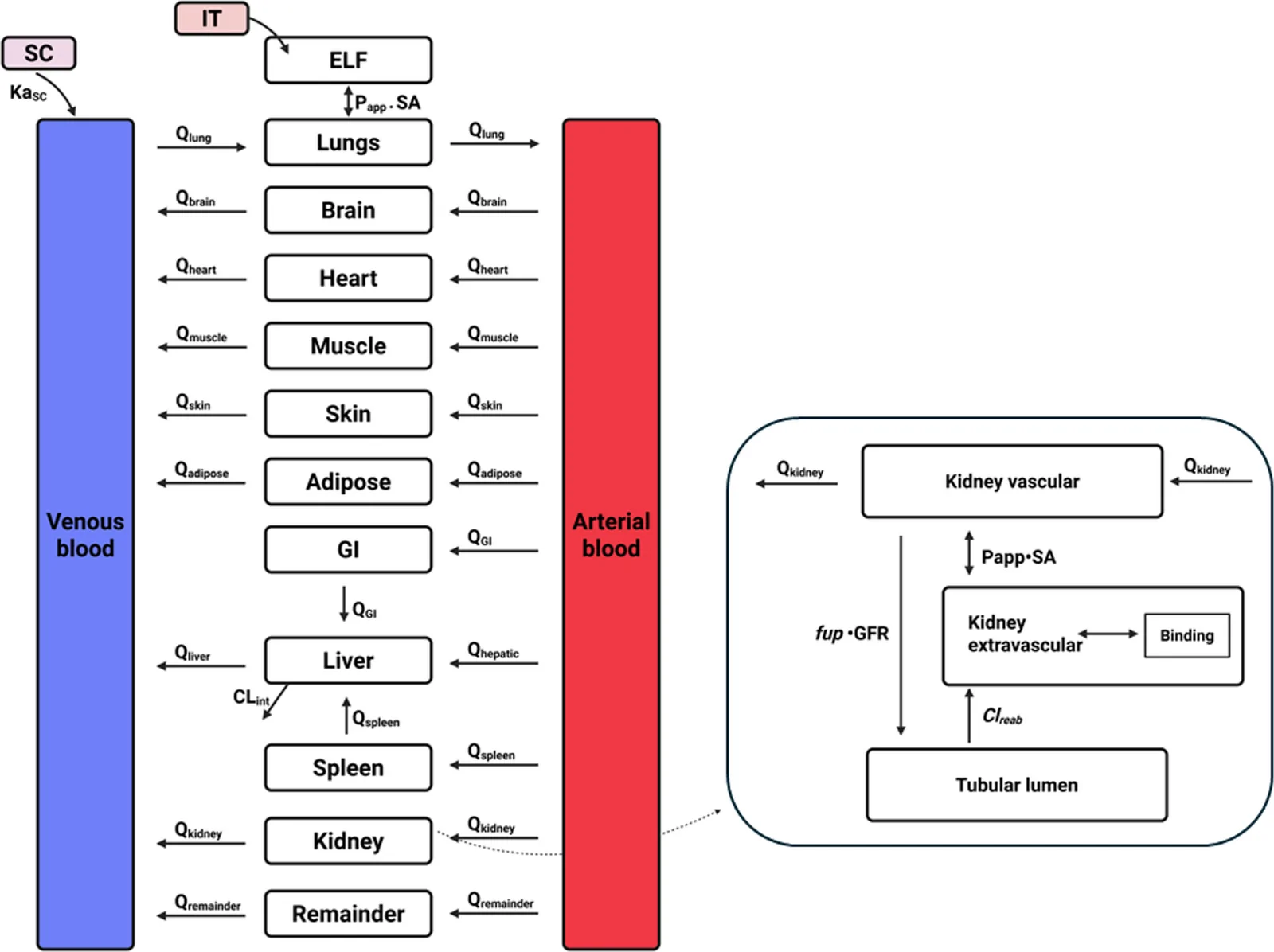
Schematic for the whole body PBPK model for polymyxin B following intratracheal (IT) and subcutaneous (SC) administration. Each tissue is represented as a perfusion-limited compartment, and the kidney is divided into vascular, tubular lumen, and extravascular sub-compartments to capture reabsorption and accumulation of polymyxin B. All physiological and model parameters are summarized in Table S1 and Table 1, respectively. *Q*_*org*_: tissue blood flow; *SC*: subcutaneous administration; *IT*: intratracheal administration; *Ka*_*SC*_: absorption rate constant; *P*_*app*_: apparent permeability; *SA*: surface area of the lung or kidney; *Cl*_*int*_: intrinsic clearance; *fup*: fraction unbound in plasma; *GFR*: glomerular filtration rate; *Cl*_*reab*_: tubular reabsorption clearance.

The model incorporated two blood compartments (venous and arterial) and ten organ compartments (e.g., lung, liver, kidney), with remaining tissues grouped into a "Remainder" compartment to ensure mass balance (Fig. 1). Physiological parameters, including organ volumes and blood flow rates, were obtained from the literature (Table S1). Cardiac output was corrected using hematocrit values to calculate total plasma flow [18]. Drug distribution was assumed to be perfusion-limited in all organs except kidney. Tissue-to-plasma partition coefficients (*Kp*) for individual organs were calculated using the Rodgers and Rowland method, which incorporates drug physicochemical properties, including *logP*, *pKa*, the unbound fraction in plasma (*fup*), and blood-to-plasma ratio (Table S2) [19]. Where necessary, calculated *Kp* values were optimized during model development using a scaling factor estimated using observed tissue concentration data. The *Kp* value for the 'Remainder' compartment was estimated from the data. All available drug and physiology-related parameters were fixed to literature values; only unknown parameters were estimated from rabbit PK data using a middle-out approach.

To account for the extensive tubular reabsorption of polymyxins, the kidney was represented by three sub-compartments: vascular, tubular lumen, and extravascular (comprising the interstitial and cellular spaces). PMB enters the tubular lumen via glomerular filtration rate (GFR) and is subsequently reabsorbed into extravascular space. Tubular reabsorption clearance (*Cl*_*reab*_) was set to nine times the urinary flow rate based on a prior colistin PBPK model and a perfused kidney study showing > 90% reabsorption of colistin [18]. Passive diffusion between the vascular and extravascular kidney compartments, defined by the product of the model-estimated apparent permeability (*Papp*_*kidney*_) and the surface area of the proximal tubular cells (*SA*_*kidney,PTC*_), along with non-specific binding in the extravascular compartment (*K*_*bind*_), was incorporated to account for the prolonged renal accumulation of PMB.

IT administration was represented as direct input into the ELF compartment linked to the lung via a permeability-limited process defined by *in vitro* apparent permeability (*Papp*_*lung*_) and lung surface area (*SA*_*lung*_). SC dosing was represented using a depot compartment with first-order absorption. The absorption rate constant (*Ka*_*SC*_) and plasma clearance (*Cl*_*p*_) were estimated by fitting SC plasma concentration–time data to a one compartment model. Renal clearance was calculated as the product of plasma clearance and the fraction of dose excreted unchanged in urine (*fe*) (Eq. 1), while non-renal clearances were determined using Eqs. (2) and (3):

1 $$C{l}_{renal}=C{l}_{p}\cdot fe$$

2 $$C{l}_{hep}=C{l}_{p}\cdot \left(1-fe\right)$$

3 $$C{l}_{int}=\frac{C{l}_{hep}\cdot {Q}_{liv}}{\left({Q}_{liv}-C{l}_{hep}\right)\cdot fup}$$

where *Cl*_*hep*_ is hepatic clearance, *Cl*_*int*_ is intrinsic clearance, and *Q*_*liv*_ is liver blood flow.

The PBPK model was first developed using SC PK data, assuming 100% bioavailability following SC administration based on previously published studies [20, 21]. When fitting the IT data, only the mucociliary clearance in the ELF compartment (*Cl*_*ELF*_) was estimated, while all other remaining parameters were fixed at the estimates obtained from fitting the SC PK data. Residual unexplained variability was assessed using additive, proportional, and combined error models. Since the combined error model provided the best fit, it was selected as the final residual error model (Eq. 4):

4 $${C}_{obs,k}= {\widehat{c}}_{pred,k}*\left( 1+ {\varepsilon}_{prop,k}\right)+ {\varepsilon}_{add,k}$$

where ${C}_{obs}$ and ${\widehat{c}}_{pred}$ are the observed and model-predicted polymyxin B concentrations for the k^th^ observation, respectively, and ${\varepsilon}_{prop,k}$ and ${\varepsilon}_{add,k}$ denote the proportional and additive error terms, respectively. Model performance was assessed via visual inspection of goodness-of-fit plots, parameter precision, physiological plausibility, and internal validation using visual predictive checks (VPC). Additional information regarding calculation of the unbound fraction in the ELF and the model equations are provided in the Supplementary Materials.

Data were analyzed using NONMEM 7.5 (ICON Development Solutions, Dublin, Ireland) using the first-order conditional estimation method with interaction (FOCE-I). Pirana was used for modeling workflow management, PKanalix (version 2024R1, Lixoft SAS, Antony, France) for non-compartmental analysis, and R (v4.2.1) for data visualization.

#### Human PBPK Model and Interspecies Scaling

To support clinical translation, the rabbit PBPK model was scaled to humans using a stepwise validation approach. The human model was validated against clinical PK data from five published studies, including plasma concentration–time profiles following IV monotherapy from three studies in healthy volunteers and critically ill patients, as well as plasma and ELF concentration–time profiles following IV and nebulized administration from two additional studies (Table S3). Physiological parameters, including organ volumes and blood flows were calculated using study-specific demographics (e.g., age and body weight) based on established equations. Other physiological parameters such as surface areas, were obtained from literature (Table S4). Tissue-to-plasma partition coefficients (*Kp*) and other drug-specific parameters were fixed to values derived from the rabbit PBPK model, while plasma clearance and mucociliary clearance were allometrically scaled from rabbit to human.

Simulations were conducted using demographic characteristics and dosing information reported for each study as detailed in Table S3. A stepwise validation strategy was applied: first, the model was assessed against IV monotherapy plasma data without parameter optimization, confirming adequate predictive performance. Subsequently, to characterize the observed ELF concentrations following IV and nebulized administration, key lung-specific parameters were optimized using one nebulization dataset. The optimized parameters were then prospectively validated against an independent nebulization dataset.

Model predictive performance was assessed by comparing simulated and observed PK parameters, AUC and C_max_ using the geometric mean fold error (GMFE) as defined in Eq. 5.

5 $$GMFE={10}^{X} \,with\, X=\frac{1}{n}{\sum}_{i=1}^{n}|{\mathrm{log}}_{10}\left(\frac{P{K}_{sim}}{P{K}_{obs}}\right)|$$

where n is the number of observations, and the ratio represents the simulated to observed PK parameter value (AUC or C_max_). A GMFE value of ≤ 2 was considered a criterion for adequate model performance.

### Identifying Efficacy and Toxicity Thresholds

To identify the optimal regimen, both efficacy and safety were considered. The plasma PK/PD target for efficacy was defined as *f*AUC_plasma_/MIC ≥ 10 [14]. ELF PK/PD target were expressed as AUC_ELF_/MIC ratios associated with antibacterial activity (1-log_10_ kill) against relevant pathogens. For *Pseudomonas aeruginosa,* a target range of 1453–1922 was applied [22]. As no PMB-specific ELF targets have been established for *Acinetobacter baumannii* and *Klebsiella pneumoniae,* colistin derived targets corresponding to 1-log_10_ kill were applied given the similar *in vitro* PD activity of both drugs: 430–1,213 for *A. baumannii* and 597 for *K. pneumoniae* [23]*. *Where a target range was available, the lower bound was used for PTA analysis to represent a conservative efficacy threshold.

Lung toxicity was defined by an ELF Cmax_ss_ threshold of 1302 mg/L based on an *in vitro* study in A549 cells [24]. Kidney toxicity was assessed using clinical nephrotoxicity data from a population PK/TD study [14], where 62 of 117 participants developed acute kidney injury (AKI). PBPK simulated PK parameters for all 117 subjects were compared between AKI and non-AKI groups, and predictive performance was assessed by receiving operating characteristics (ROC) analysis with optimal thresholds determined by the Youden index.

### Monte Carlo Simulations

Monte Carlo simulations (n = 1,000) were performed to estimate probability of target attainment (PTA) and probability of toxicity attainment (PToXA) across IV and nebulization regimens for pathogens with varying MICs. IV regimens included loading doses of 2 mg/kg, 2.5 mg/kg, or 150 mg with maintenance doses of 0.5 mg/kg, 1.25 mg/kg, 1.5 mg/kg, 75 mg, or 100 mg every 12 h (1 h infusion). Nebulization regimens included 25, 50, or 75 mg every 12 h administered over 15 min, evaluated as monotherapy and in combination with IV therapy. Virtual subjects demographics were derived from the largest available clinical trial dataset [25], with physiological parameters calculated using established equations (Table S4). PTA was evaluated at steady state, and regimens were considered optimal when PTA ≥ 90% and PToXA < 40%.

### Mechanism-based PK/PD Model

Static concentration time-kill (SCTK) assays were performed to evaluate the efficacy of PMB against two *A.baumannii* strains (ATCC19606 and Ab124). Data from SCTK were used to develop MBM, with full experimental and modeling details provided in the *Supplementary Methods*. PBPK-simulated unbound PMB ELF concentrations served as the PK driver for bacterial killing in MBM. The integrated PBPK-MBM framework predicted bacterial load reductions under IV monotherapy (loading dose: 2.5 mg/kg + maintenance dose: 0.5 mg/kg every 12 h), inhalation (INH) monotherapy (75 mg every 12 h), and IV + INH combination therapy.

## Results

### Rabbit PBPK Model

**Fig. 2:**
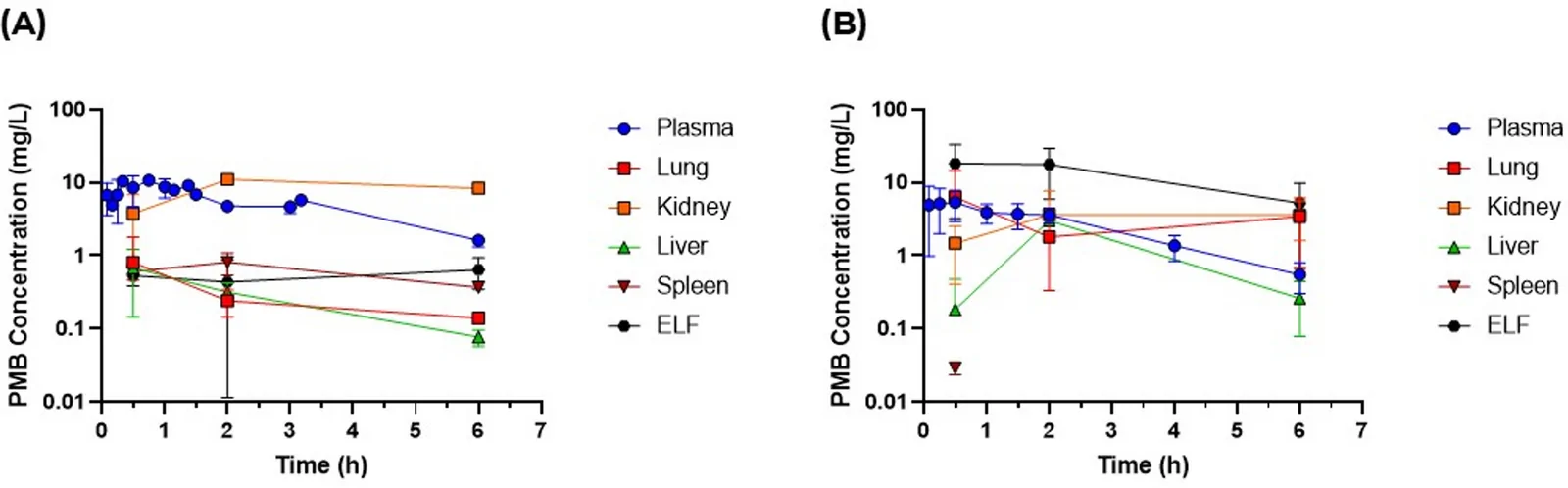
*In vivo* pharmacokinetics of polymyxin B (PMB: 2 mg/kg) following subcutaneous (**A**) and intratracheal (**B**) administration in rabbits. PMB concentrations were quantified in plasma, epithelial lining fluid (ELF) and homogenized tissues (lung, kidney, liver and spleen). Three rabbits were euthanized at each timepoint (0.5, 2 and 6 h) to obtain ELF and tissue samples.

The observed mean PMB PK profiles in plasma, ELF, lung, liver, kidney, and spleen following SC and IT administration are shown in Fig. 2, and the associated non-compartmental PK parameters are summarized in Table S5. PMB distribution to the kidney following IT administration was assessed by immunofluorescence imaging; PMB was detected in the renal cortex of both kidneys within 30 min of dosing (Figure S1A), confirming rapid transfer from the lungs to the systemic circulation. A similar distribution was observed 6 h post-treatment (Figure S1B).

**Fig. 3:**
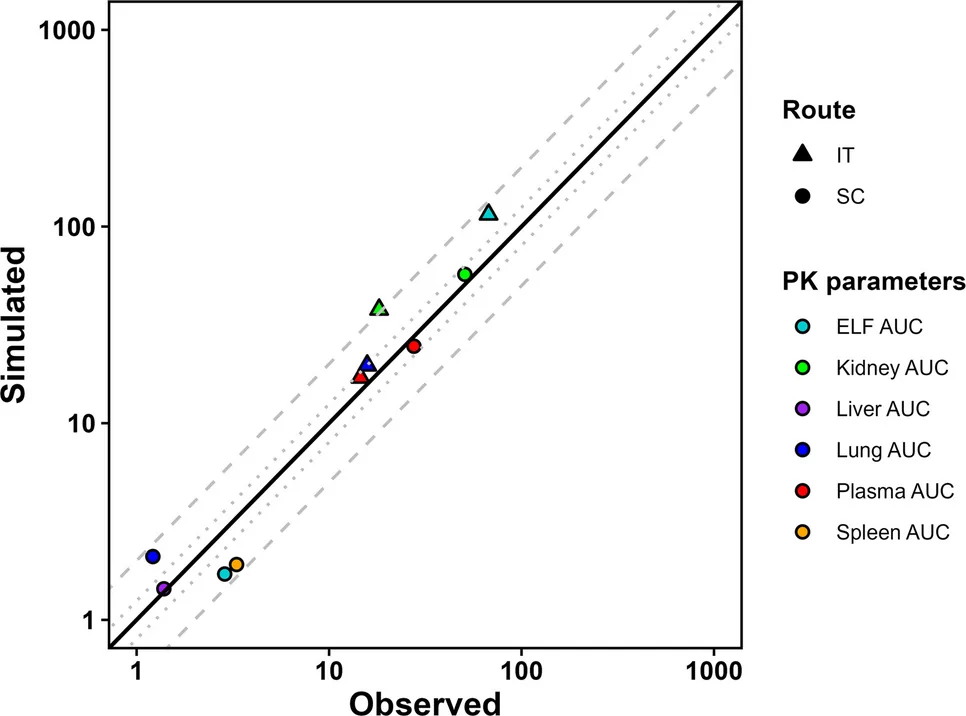
Goodness-of-fit plot comparing simulated *versus* observed pharmacokinetic metrics (AUC, mg·h/L) for polymyxin B (2 mg/kg) administered subcutaneously (SC) or intratracheally (IT) in rabbits. The solid line represents the line of identity, while the dotted and dashed lines indicate 1.25-fold and 2-fold deviation boundaries, respectively.

Model-based analysis estimated a first-order absorption rate constant (*Ka*_*SC*_) of 6.55 h^−1^. Estimated plasma (*Cl*_*p*_) and mucociliary (*Cl*_*ELF*_) clearances were 0.18 L/h and 0.02 L/h, respectively. Of the total plasma clearance, renal clearance accounted for 0.01 L/h, while non-renal clearance was 0.17 L/h. Although tissue to plasma partition coefficients (*Kp*) calculated using Rodgers and Rowland method adequately described observed concentrations in most organs, a scaling parameter was required to optimize model fits for the lung and ELF compartments. Final PBPK model parameters are summarized in Table 1. A combined residual error model was selected, which accounts for both proportional and additive components of unexplained variability in the observed concentration data. Model-derived AUC were within a two-fold deviation of observed values (Fig. 3), and simulated PK profiles adequately captured the observed data in plasma and all sampled tissues following SC administration (Figure S2A). For IT administration, plasma concentrations were well characterized; however, tissue concentrations were slightly overpredicted at some time points, with predictions at the margins of two-fold acceptability in some tissues (Figure S2B). Although a more complex lung compartment model incorporating vascular, endothelial and interstitial regions was explored, it was not identifiable from the limited tissue observations available, and the current structure represented the best achievable fit to the data.

**Table 1:** Rabbit PBPK model parameters

Parameter	Value (RSE%)	Reference
Absorption rate constant after SC (*Ka*_*SC*_) (hr ^−1^)	6.55 (16.3)	Estimated
Plasma clearance (*Cl*_*p*_) (L/h)	0.18 (5.4)	Estimated
Tubular reabsorption clearance of polymyxin B (*Cl*_*reab*_) (L/h)	0.06	[17] Equation (1)^a^
Apparent permeability in lung (*Papp*_*lung*_) (Cm/s)	9.33·10^–6^	[43]
Surface area of lung (*SA*_*lung*_) (Cm^2^)	59,330	[44]
Permeability rate between Lung and ELF (L/h)	1.99	Equation (2)^a^
Tissue to plasma partition coefficient for remainder compartment (*Kp*_*rem*_)	0.4 (8.7)	Estimated
Binding rate constant of PMB in renal tissues (*K*_*bind*_) (hr ^−1^)	0.091	[18]
Unbinding rate constant of PMB in renal tissues (*K*_*unbind*_) (hr ^−1^)	0.0145	[18]
Apparent permeability in kidney (*Papp*_*kidney*_) (Cm/s)	6.02 ·10^–7^ (3%)	Estimated
Surface area of kidney proximal tubular cells (*SA*_*kidney,PTC*_) (Cm^2^)	133200	[45]
Passive diffusion between renal blood flow and tissues (L/h)	0.60	Equation (3)^a^
Scalar of tissue to plasma partition coefficient of lung (*S*_*Kp,lun*_)	0.67 (15.2)	Estimated
Scalar of unbound tissue to plasma partition coefficient of lung (*S*_*Kpu,lun*_)	6.68 (16.5)	Estimated
Additive residual error model (mg/L)	0.08 (32.1)	Estimated
Proportional residual error model	0.2 (21.1)	Estimated
**IT**		
Mucociliary clearance from ELF (*Cl*_*ELF*_) (L/h)	0.02 (12.7)	Estimated
Scalar of tissue to plasma partition coefficient of lung (*S*_*Kp,lun,IT*_)	10.1 (1)	Estimated
Scalar of unbound tissue to plasma partition coefficient of lung (*S*_*Kpu,lun,IT*_)	0.92 (2.7)	Estimated
Additive residual error model (mg/L)	2.04 (27.8)	Estimated
Proportional residual error model	0.33 (18.7)	Estimated

^a^Equation (1): Cl_reab_ = 9 · urine flow; Equation (2): Permeability rate between Lung and ELF = *Papp*_*lung*_ · *SA*_*lung*_ · 3.6; Equation (3): Passive diffusion between kidney vascular and extravascular compartments = *Papp*_*kidney*_ ·*SA*_*kidney,PTC*_ ·3.6

Using the final rabbit PBPK model, lung and kidney concentrations were simulated for both SC and IT administration to compare the total systemic and site-specific exposure (AUC_0–6 h_). IT administration resulted in a ~ 10.4-fold increase in lung exposure relative to SC dosing (25.3 *vs.* 2.43 mg·h/L), while kidney exposure was reduced by ~ 27.7% (45.9 *vs.* 63.5 mg·h/L), highlighting the potential of inhaled PMB to enhance local pulmonary efficacy while attenuating renal accumulation.

### PBPK Model Translation to Humans

**Fig. 4:**
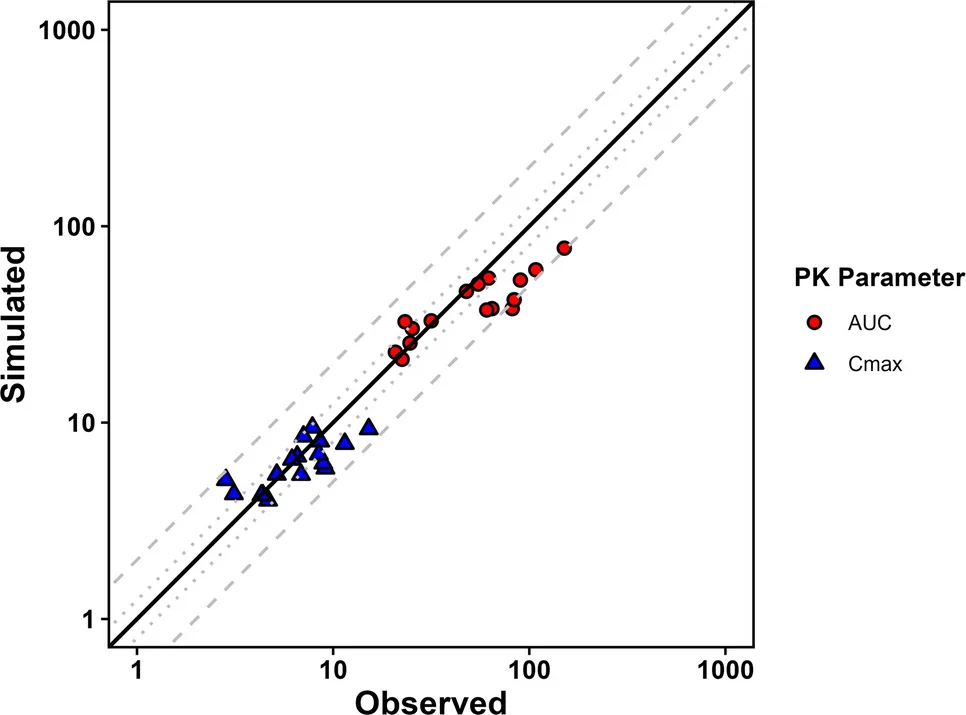
Goodness-of-fit plot comparing simulated *versus* observed pharmacokinetic metrics (AUC,mg·h/L and C_max_,mg/L) for polymyxin B administered intravenously in healthy volunteers and critically ill patients (*n* = 17). The solid line represents the line of identity, while the dotted and dashed lines indicate 1.25-fold and 2-fold deviation boundaries, respectively.

The rabbit PBPK model was successfully extrapolated to humans by incorporating study-specific demographics and allometric scaling. For a critically ill patient population with an average body weight of 75.7 kg, the allometrically-scaled plasma clearance was estimated at 1.97 L/h. The human PBPK model was validated by adequately predicting plasma PK parameters in healthy volunteers and critically ill patients receiving IV dosing (n = 17), with AUC and C_max_ values within two-fold of observed data (Fig. 4). GMFE values were 1.39 for AUC (observed: 59.5 ± 35.6 *vs.* simulated: 41.5 ± 14.6 mg·h/L) and 1.25 for C_max_ (observed: 7.09 ± 3.04 *vs.* simulated: 6.36 ± 1.71 mg/L), both within the acceptable threshold of ≤ 2. Individual simulations demonstrated adequate capture of the observed plasma concentrations over time profiles (Figure S3).

**Fig. 5:**
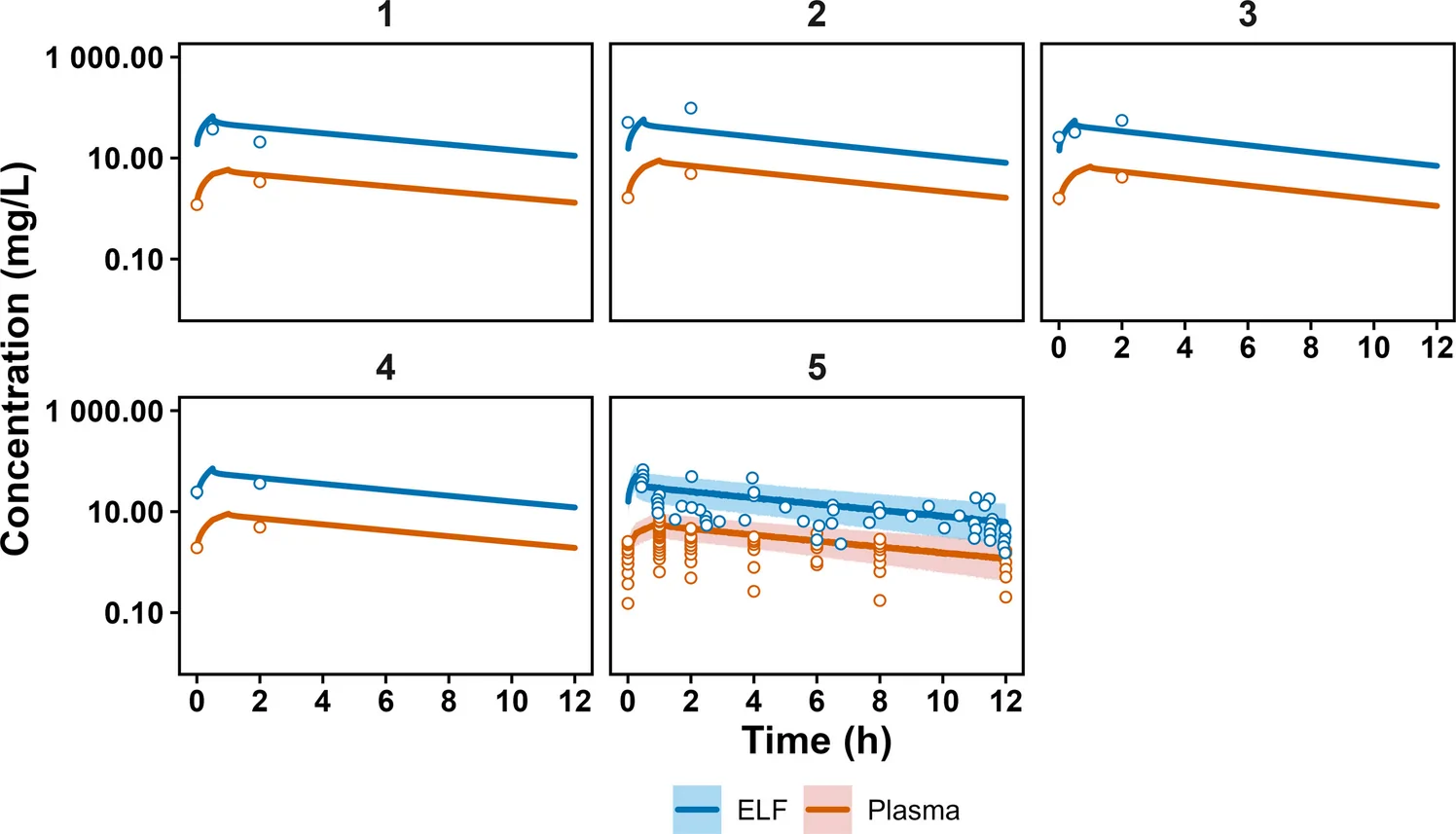
PBPK model-based simulations of polymyxin B concentrations in plasma and epithelial lining fluid (ELF) for critically ill patients receiving intravenous and nebulized polymyxin B. Solid lines represent median model simulated concentrations, shaded regions depict the 90% prediction interval, and open circles denote observed concentrations. Observed data for Subjects 1—4 correspond to individual patient data from Liu *et al*., 2025 and Subject 5 represents population-level data from Li *et al*., 2025.

The model was subsequently validated for pulmonary delivery using datasets from two clinical studies that reported both plasma and ELF concentrations following IV and nebulized administration. To accurately characterize the observed ELF concentrations, the lung specific unbound tissue-to-plasma partition coefficient scaling parameter (*S*_*Kpu,lun,IT*_) and *CL*_*ELF*_ were optimized to 0.25 and 0.01 L/h, respectively. The model adequately described plasma and ELF concentration–time profiles at both individual and population levels (Fig. 5). For individual-level validation, simulated plasma AUC values for four subjects from Liu *et al*. ranged from 71–109 mg·h/L, aligning with the reported observed range of 56.7–85.7 mg·h/L. Simulated ELF AUC ranged from 492–714 mg·h/L, which were also consistent with reported range of 284–1,873 mg·h/L [26]. For population-level validation, the simulated median plasma AUC of 35.5 mg·h/L was consistent with the reported median of 35 mg·h/L (IQR: 22.0–54.2 mg·h/L). The simulated median ELF AUC of 203 mg·h/L was higher than the reported median of 52.5 mg·h/L (IQR: 35.3–102.8 mg·h/L), although it remained within twofold of the upper quartile, reflecting the high variability in ELF PK [27].

It should be noted that the comparison between simulated and observed AUC values are limited by the available published data; Liu *et al*. derived AUC_24h_ from only two plasma samples per subject, and Li *et al*. did not report the method used for AUC calculation. These limitations should be considered when interpreting the differences between simulated and reported values.

### ROC Analysis

The human PBPK model was used to simulate plasma PK profiles of 117 subjects with their individual demographics such as GFR from a PK/TD study. The majority of simulated AUC values were within twofold of observed data in both AKI and non-AKI patients (Figure S4). Simulated PK parameters were compared between the AKI and non-AKI groups (Table S6), and ROC curve analysis was performed to identify PK parameters associated with higher risk of AKI onset (Figure S5). Both AUC_SS,24 h,plasma_ and *f*AUC_SS,24 h,plasma_ yielded an area under ROC curve of 0.7, indicating moderate-to-good predictive performance. An optimal threshold of 92.9 mg⋅h/L for AUC_SS,24 h,plasma_ yielded a sensitivity of 63.8% and a specificity of 64%, suggesting that patients exceeding this exposure level may be at higher risk of developing AKI. Kidney tissue exposure (AUC_SS,24 h,kidneyev_) was also showed moderate discriminatory ability (ROC AUC = 0.6), with a threshold of 157 mg·h/L providing a sensitivity of 70.7% and specificity of 56%.

### Monte Carlo Simulations

**Fig. 6:**
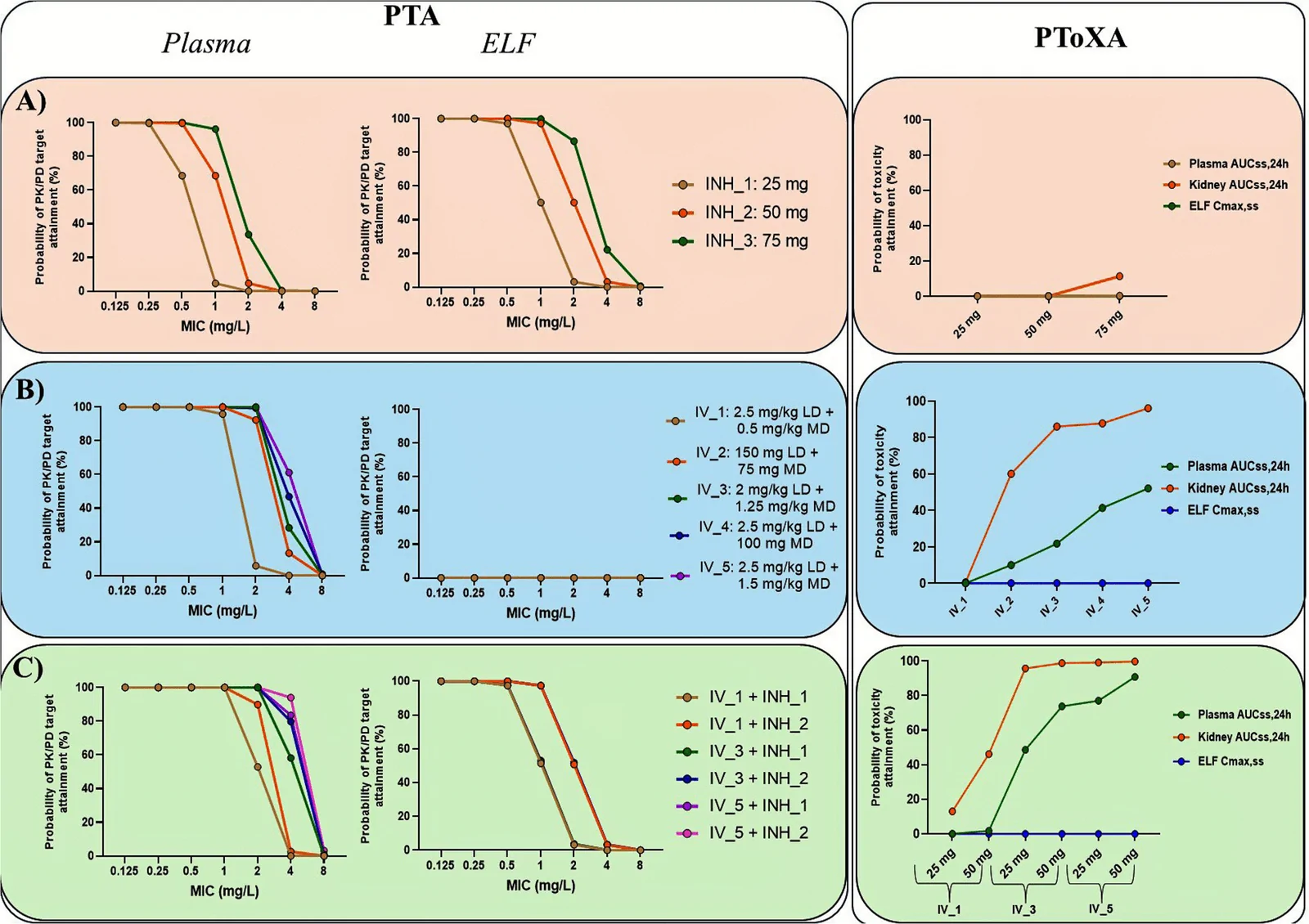
Probability of PK/PD target attainment (PTA) in plasma and epithelial lining fluid (ELF) and probability of toxicity attainment (PToXA) in plasma, kidney, and ELF following (**A**) inhalation (INH) monotherapy, (**B**) intravenous (IV) monotherapy, (**C**) combined IV + INH therapy against *Acinetobacter baumannii.*

Monte Carlo simulations were performed to evaluate the PTA and PToXA for INH and IV PMB. Virtual subjects (*n* = 1000) represented a wide range of clinical covariates including creatinine clearance (60–120 ml/min), age (16–94 years), and body weight (34–164 kg). Simulations were performed against MIC values of 0.125–8 mg/L for *A. baumannii* (Fig. 6), *K. pneumoniae* (Figure S6), and *P. aeruginosa* (Figure S7).

None of the evaluated IV monotherapy regimens achieved the required PTA in ELF, despite attaining ≥ 90% PTA in plasma for MICs up to 1 mg/L. Among five IV maintenance regimens, two (MD: 1.5 mg/kg, and 100 mg) exhibited an unfavorable safety profile, with ≥ 40% PToXA in both plasma and kidney. Regimen with MD of 1.25 mg/kg showed 21.8% and 86% PToXA in plasma and kidney respectively. Notably, no pulmonary toxicity was observed following IV administration.

In contrast, INH monotherapy achieved favorable PTA in both plasma and ELF for all three pathogens, while maintaining a < 15% PToXA. While combined IV and INH slightly improved plasma PTA, it resulted in minimal changes to ELF PTA compared to INH monotherapy. Although the cumulative PToXA was higher for IV + INH combinations, there was no statistically significant difference (p > 0.05) in toxicity between combination regimens using clinical INH doses (25 mg and 50 mg) and IV administration alone.

### Bacterial Load Reduction in Critically Ill Patients

**Fig. 7:**
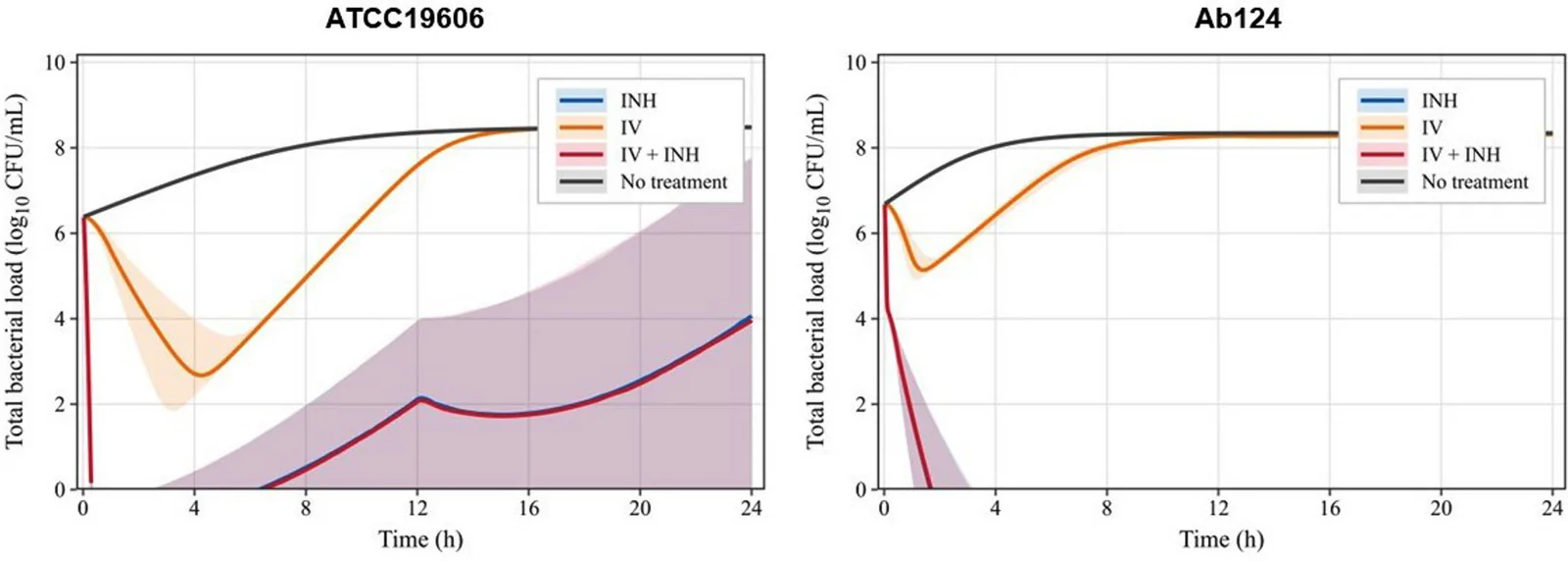
PBPK-MBM model-predicted bacterial count profiles following treatment with polymyxin B as intravenous (IV) monotherapy, inhalation (INH) monotherapy, or combined IV + INH therapy against two *A. baumannii* isolates. The IV regimen consisted of a 2.5 mg/kg PMB loading dose followed by a 0.5 mg/kg every 12 h maintenance dose. The INH regimen consisted of 75 mg every 12 h. Solid lines represent median model predictions, and shaded regions depict the 90% prediction intervals based on simulations with 1000 virtual patients.

The MBM adequately characterized the SCTK data, as shown in Figure S8-9. The optimal regimen selected for the simulation was based on a PToXA  < 40%. Integrated PBPK-MBM simulations utilizing the simulated ELF PK concentrations to drive the pharmacodynamic response indicated that IV monotherapy had very minimal bactericidal activity, followed by complete regrowth within 24 h. In contrast, inhaled monotherapy achieved a robust reduction in bacterial load (≥ 4 log_10_ CFU/mL) at 24 h against both PMB-susceptible (ATCC19696, PMB MIC: 0.5 mg/L) and PMB-resistant (Ab124, PMB MIC: 4 mg/L) *A. baumannii* isolates. Notably, combined IV and INH provided no additional bactericidal benefit over INH monotherapy (Fig. 7), suggesting that the local ELF concentrations achieved with INH are the primary determinants of antimicrobial efficacy.

## Discussion

This study provides one of the first quantitative assessments of PMB plasma and tissue distribution following IT administration in rabbits, from which a whole-body PBPK model was developed, extrapolated to humans and validated against clinical PK data. IT administration of aerosolized PMB achieved ~ 10.4-fold higher lung exposure relative to SC, while reducing kidney exposure by 27.7%. Monte Carlo simulations demonstrated that PMB INH monotherapy may achieve favorable PTA in both plasma and ELF for all three pathogens evaluated, whereas IV monotherapy yielded 0% PTA in ELF. Integrated PBPK-MBM simulations suggested that inhaled monotherapy may produce substantial bacterial load reduction (≥ 4 log_10_ CFU/mL) against both PMB-susceptible and PMB-resistant *A. baumannii*, while IV monotherapy was associated with minimal bactericidal activity followed by complete regrowth within 24 h. Combined IV plus INH regimens did not appear to confer additional bactericidal benefit over INH monotherapy.

While inhaled colistin is well-established for respiratory tract infections [28–31], there remains a substantial gap in PK/PD data to guide the dosing of inhaled PMB. Only one published study has characterized PMB exposure in plasma and ELF following aerosolized administration, and that was conducted in mice [22]. Prior PBPK modeling efforts for PMB relied solely on plasma concentrations after IV dosing, without tissue-level time-course data [32]. Our model represents a substantive advance, incorporating plasma, lung, kidney, liver, and spleen data following both systemic and direct pulmonary delivery, and to our knowledge, this is the first PBPK model to characterize the PMB tissue distribution after IT dosing. The rabbit model was essential for this purpose, as it provided the organ-level distribution parameters that informed the human model and enabled tissue-level predictions.

Consistent with established colistin models, perfusion-limited distribution was assumed for all organs except the kidney, which was divided into sub-compartments to capture PMB accumulation via reabsorption. Although saturable uptake kinetics have been reported in renal proximal tubular cells study, the Michaelis–Menten constant (Km = 13 mg/L) exceeds the kidney concentrations observed in this study [33], and colistin data indicate no renal saturation at conventional doses [34]. Hence, a linear reabsorption rate constant was implemented. With less than 5% of the administered dose recovered in urine, non-renal pathways appear to dominate. *In vitro* data suggest that hepatic metabolism mediated by unidentified (non-CYP) enzymes is a key clearance pathway, with limited evidence of biliary excretion [35].

Tissue distribution of PMB is governed by protein-binding to α−1-acid glycoprotein and human serum albumin [32, 36], as well as partition coefficients. The optimized lung partition coefficient (Kp_lun =_ 0.075) suggests limited pulmonary distribution following systemic delivery, consistent with previously reported low plasma-to-ELF intercompartmental clearance (0.03 L/h) in humans [27]. Similar findings have been observed in mice, where a low transfer rate from plasma to ELF (1.14 × 10⁻^4^ L/h/kg) resulted in negligible ELF exposure following intravenous administration. Together, these observations support the limited pulmonary penetration of systemically administered polymyxin B [22]. In contrast, IT administration achieved markedly higher ELF and lung exposure with substantially reduced systemic exposure.

Accordingly, route-specific scaling factors were required to adequately describe both administration routes within a single model structure, suggesting that the underlying distribution mechanisms differ between systemic and direct pulmonary delivery. Transport of PMB from the lungs to the systemic circulation may be facilitated by peptide transporter PEPT2 expressed in lung tissue [11], however, the quantitative contribution of this pathway remains unclear [11]. Similarly, renal accumulation is thought to occur through megalin-mediated endocytosis and transporters such as PEPT2 and OCTN2, but the relative contributions of these mechanisms have yet to be fully elucidated [14].

Our simulations showing that INH regimens achieved favorable ELF PTA while IV monotherapy yielded 0% ELF PTA are consistent with clinical studies of pneumonia caused by CR-GNB, which reported no significant differences in clinical or microbiological cure or mortality between INH-only and IV + INH groups [10]. Furthermore, comparative studies of IV monotherapy *versus* IV + INH PMB have shown no significant difference in nephrotoxicity rates (5.2% for IV + INH *vs.* 4.6% for IV alone) [37, 38]. Consistent with these data, our model-based simulations revealed no substantial difference in the nephrotoxicity risk between IV and IV + INH regimens, as renal impairment is primarily driven by systemic exposure.

A key strength of this study is the successful extrapolation and validation of the PBPK model from rabbits to humans. Allometrically-scaled plasma clearance (1.22–2.96 L/h) aligned well with reported human values (0.49—4.24 L/h) [25], and simulations adequately captured both individual and population-level plasma and ELF concentration–time profiles, with simulated exposures within the two-fold of observed values. The integration of distinct lung and kidney toxicity thresholds, derived from *in vitro* and clinical PK/TD dataset allowed simultaneous assessment of efficacy and safety, a methodological advance over models relying solely on plasma PTA. The use of 3Rs-compliant study designs with sparse tissue sampling in USDA-protected species further demonstrates that robust translational PBPK models can be developed from constrained datasets.

ROC analysis identified a plasma AUC_SS,24 h_ of 92.9 mg·h/L and a kidney exposure (AUC_SS,24 h,kidneyev_) threshold of > 157 mg·h/L as associated with higher risk of AKI onset. These thresholds should be interpreted as indicators of elevated AKI risk rather than definitive predictors of nephrotoxicity, given the moderate sensitivity and specificity observed, and require further validation in prospective clinical studies. The plasma threshold is consistent with previously reported values (~ 100 mg·h/L), supporting the plausibility of our model predictions [14]. While previous PK/TD models primarily linked plasma exposure to AKI, the present approach additionally identified kidney tissue concentrations associated with renal impairment, providing a more mechanistically grounded basis for nephrotoxicity assessment [14].

However, few limitations warrants further consideration. Due to the absence of rabbit-specific plasma protein binding data, we assumed protein binding equivalent to humans, given similar α−1-acid glycoprotein and serum albumin levels reported in the two species [39]. PMB also binds to pulmonary surfactants, proteins, and mucins within ELF. Based on evidence indicating that ELF protein concentrations are ~ 60% of plasma levels [40], we assumed 45% ELF binding. Although previous colistin PBPK models assumed higher binding rates (up to 85%), the extent of PMB binding in human ELF remains uncertain. While most PK/PD targets reference total ELF exposure, future research should prioritize direct quantification of PMB binding in ELF to refine dose–response relationships. Additionally, due to sparse tissue sampling, more complex lung compartment models such as those incorporating central and peripheral regions with distinct vascular, endothelial, and interstitial spaces could not be reliably identified from the available data. Future studies employing microdialysis sampling in specific lung regions would enable site-specific estimation of unbound concentrations and support the development of more physiologically detailed lung models, including transporter-mediated processes such as PEPT2 [11]. Although the current experimental design did not permit incorporation of physiological changes associated with pulmonary infection, the PBPK framework provides a flexible platform for integrating disease-related alterations as relevant data become available. These may include changes in ELF composition (e.g., protein content and pH), increased alveolar-capillary permeability, inflammatory edema, surfactant dysfunction, and altered transporter expression [11]. Incorporation of these factors will enhance the physiological relevance of the model, improve prediction of pulmonary PMB exposure under diseased conditions, and strengthen its utility for optimizing antibacterial therapy in patients with pulmonary infections.

With respect to pulmonary safety, INH PMB monotherapy was associated with high tolerability and rare discontinuations due to airway reactions [10]. The most common adverse event was bronchospasm, typically infrequent and mild, which has a cumulative incidence of < 5% [5]. Although *in vitro* exposure to 1 mM PMB (1301.56 mg/L) induced cytotoxic effects in A549 cells [24], this concentration exceeds the ELF C_max_ predicted by our model, suggesting that clinically relevant inhaled PMB exposures are unlikely to reach cytotoxic thresholds. In the absence of an established *in vivo* pulmonary toxicity threshold for PMB, this *in vitro* benchmark was used as a conservative reference point.

Taken together, these findings suggest that INH PMB monotherapy may represent as a strategy to maximize pulmonary efficacy while limiting systemic toxicity, though prospective clinical validation is required. The success of this approach is highly dependent on the delivery technique; vibrating mesh nebulizers are preferred because they achieve high lung deposition efficiencies (40–60%) with minimal residual drug following administration [22]. Although current IDSA guidelines recommend the use of concomitant inhaled and systemic antibiotics for HAP/VAP caused by Gram-negative pathogens, systemic therapy can be strategically optimized to reduce the risk of nephrotoxicity. Specifically, INH PMB may be paired with non-polymyxin intravenous agents such as β-lactam/β-lactamase inhibitor combinations, which provide adequate pulmonary penetration while maintaining a favorable systemic safety profile. Consistent with this strategy, clinical studies evaluating INH polymyxin monotherapy frequently employed with β-lactam/β-lactamase inhibitor combinations as systemic adjuncts, reported favorable clinical outcomes [41, 42]. This treatment paradigm leverages high local pulmonary exposure while preserving systemic safety. Prospective clinical trials are now needed to validate this approach and establish a safer, more effective standard of care. By quantifying lung exposure and explicitly integrating efficacy–toxicity tradeoffs, this framework moves inhaled PMB therapy beyond empirical use toward predictive, mechanism-based dosing strategies. Collectively, these findings provide a rigorous foundation for designing future PK/PD studies that will aid in accelerating the clinical translation of optimized inhaled PMB regimens for the treatment of multidrug-resistant Gram-negative pathogens.

## Supplementary Information

Below is the link to the electronic supplementary material.

Supplementary file1 (DOCX 3.26 MB)

## Data Availability

All data that support the findings of this study are provided in the article and the Supplementary materials.

## References

[1] Zhou L, Wang D, Li X, Cheng Y, Lin Y, Weng Q, *et al*. The relationship between the alveolar epithelial lining fluid concentration of intravenous plus inhaled polymyxin B and clinical efficacy in patients with pneumonia caused by carbapenem-resistant gram-negative bacilli: a prospective cohort study. BMC Infect Dis. 2025;25:1135. 10.1186/s12879-025-11515-z. PMC12465416 41013329

[2] Zhou J, Chen M, Liang M, Han X, Weng R, Li Y, *et al*. Diverse modes of ceftazidime/avibactam resistance acquisition in carbapenem-resistant *Klebsiella pneumoniae* and *Pseudomonas aeruginosa* from a Chinese intensive care unit. Ann Clin Microbiol Antimicrob. 2025;24:35. 10.1186/s12941-025-00800-z. PMC12125794 40448249

[3] Mishra A, Dwivedi R, Faure K, Morgan DJ, Cohn J. Estimated undertreatment of carbapenem-resistant Gram-negative bacterial infections in eight low-income and middle-income countries: a modelling study. Lancet Infect Dis. 2025;25:1011–9. 10.1016/S1473-3099(25)00108-2. PMC12367590 40318677

[4] Tsuji BT, Pogue JM, Zavascki AP, Paul M, Daikos GL, Forrest A, *et al*. International consensus guidelines for the optimal use of the polymyxins: endorsed by the American College of Clinical Pharmacy (ACCP), European Society of Clinical Microbiology and Infectious Diseases (ESCMID), Infectious Diseases Society of America (IDSA), International Society for Anti-infective Pharmacology (ISAP), Society of Critical Care Medicine (SCCM), and Society of Infectious Diseases Pharmacists (SIDP). Pharmacotherapy. 2019;39:10–39. 10.1002/phar.2209. PMC7437259 30710469

[5] Almangour TA, Garcia E, Zhou Q, Forrest A, Kaye KS, Li J, *et al*. Polymyxins for the treatment of lower respiratory tract infections: lessons learned from the integration of clinical pharmacokinetic studies and clinical outcomes. Int J Antimicrob Agents. 2021;57:106328. 10.1016/j.ijantimicag.2021.106328. 33785362

[6] Almangour TA, Alruwaili A, Almutairi R, Alrasheed A, Alhifany AA, Eljaaly K, *et al*. Aerosolized plus intravenous colistin vs intravenous colistin alone for the treatment of nosocomial pneumonia due to multidrug-resistant Gram-negative bacteria: a retrospective cohort study. Int J Infect Dis. 2021;108:406–12. 10.1016/j.ijid.2021.06.007. 34111542

[7] Hussain K, Salat MS, Ambreen G, Mughal A, Idrees S, Sohail M, *et al*. Intravenous vs intravenous plus aerosolized colistin for treatment of ventilator-associated pneumonia - a matched case-control study in neonates. Expert Opin Drug Saf. 2020;19:1641–9. 10.1080/14740338.2020.1819980. 32892635

[8] Nation RL, Velkov T, Li J. Colistin and polymyxin B: peas in a pod, or chalk and cheese? Clin Infect Dis. 2014;59:88–94. 10.1093/cid/ciu213. PMC4305129 24700659

[9] Lin Y-W, Zhou QT, Cheah S-E, Zhao J, Chen K, Wang J, *et al*. Pharmacokinetics/pharmacodynamics of pulmonary delivery of colistin against *Pseudomonas aeruginosa* in a mouse lung infection model. Antimicrob Agents Chemother. 2017;61:e02025-16. 10.1128/AAC.02025-16. PMC5328518 28031207

[10] Cheng Y, Zhou L, Wang D, Li X, Lin R, Chen J, *et al*. Inhaled alone versus inhaled plus intravenous polymyxin B for the treatment of pneumonia due to carbapenem-resistant gram-negative bacteria: a prospective randomized controlled trial. Int J Antimicrob Agents. 2025;65:107483. 10.1016/j.ijantimicag.2025.107483. 40023452

[11] Yeshwante S, Mahadevan R, Sule O, Tiley JB, Luterbach CL, McCann S, *et al*. Modeling lung transporters and their influence on inhaled drug disposition. Pharmacol Rev. 2025. 10.1016/j.pharmr.2025.100100. 41297206

[12] Yeshwante SB, Hanafin P, Miller BK, Rank L, Murcia S, Xander C, *et al*. Pharmacokinetic considerations for optimizing inhaled spray-dried pyrazinoic acid formulations. Mol Pharm. 2023;20:4491–504. 10.1021/acs.molpharmaceut.3c00199. PMC10868345 37590399

[13] Fox-Dewhurst R, Alberts MK, Kajikawa O, Caldwell E, Johnson MC, Skerrett SJ, *et al*. Pulmonary and systemic inflammatory responses in rabbits with gram-negative pneumonia. Am J Respir Crit Care Med. 1997;155:2030–40. 10.1164/ajrccm.155.6.9196112. 9196112

[14] Hanafin PO, Mahadevan R, Rao GG, Zavascki AP, Sandri AM, Kwa A, *et al*. Population pharmacokinetic/toxicodynamic model for polymyxin B in critically ill patients to identify the risk of nephrotoxicity. Clin Pharmacol Ther. 2025;118:841–52. 10.1002/cpt.3729. PMC12353974 40524377

[15] Petraitiene R, Petraitis V, Kavaliauskas P, Maung BBW, Khan F, Naing E, *et al*. Pharmacokinetics and efficacy of ceftazidime-avibactam in the treatment of experimental pneumonia caused by *Klebsiella pneumoniae* carbapenemase-producing *K. pneumoniae* in persistently neutropenic rabbits. Antimicrob Agents Chemother. 2020;64:e02157-19. 10.1128/AAC.02157-19. PMC7179283 32015048

[16] Velkov T, Yun B, Schneider EK, Azad MAK, Dolezal O, Morris FC, *et al*. A novel chemical biology approach for mapping of polymyxin-lipopeptide antibody binding epitopes. ACS Infect Dis. 2016;2:341–51. 10.1021/acsinfecdis.6b00031. PMC5321607 27627202

[17] Bouchene S, Marchand S, Couet W, Friberg LE, Gobin P, Lamarche I, *et al*. A whole-body physiologically based pharmacokinetic model for colistin and colistin methanesulfonate in rat. Basic Clin Pharmacol Toxicol. 2018;123:407–22. 10.1111/bcpt.13026. 29665289

[18] Viel A, Henri J, Bouchène S, Laroche J, Rolland J-G, Manceau J, *et al*. A population WB-PBPK model of colistin and its prodrug CMS in pigs: focus on the renal distribution and excretion. Pharm Res. 2018;35:92. 10.1007/s11095-018-2379-4. 29532176

[19] Utsey K, Gastonguay MS, Russell S, Freling R, Riggs MM, Elmokadem A. Quantification of the impact of partition coefficient prediction methods on physiologically based pharmacokinetic model output using a standardized tissue composition. Drug Metab Dispos. 2020;48:903–16. 10.1124/dmd.120.090498. 32665416

[20] Landersdorfer CB, Wang J, Wirth V, Chen K, Kaye KS, Tsuji BT, *et al*. Pharmacokinetics/pharmacodynamics of systemically administered polymyxin B against Klebsiella pneumoniae in mouse thigh and lung infection models. J Antimicrob Chemother. 2018;73:462–8. 10.1093/jac/dkx409. PMC5890666 29149294

[21] Jiao Y, Yan J, Vicchiarelli M, Sutaria DS, Lu P, Reyna Z, *et al*. Individual components of polymyxin B modeled via population pharmacokinetics to design humanized dosage regimens for a bloodstream and lung infection model in immune-competent mice. Antimicrob Agents Chemother. 2023;67:e0019723. 10.1128/aac.00197-23. PMC10190254 37022153

[22] Lin Y-W, Zhou Q, Onufrak NJ, Wirth V, Chen K, Wang J, *et al*. Aerosolized polymyxin B for treatment of respiratory tract infections: determination of pharmacokinetic-pharmacodynamic indices for aerosolized polymyxin B against *Pseudomonas aeruginosa* in a mouse lung infection model. Antimicrob Agents Chemother. 2017;61:e00211-17. 10.1128/AAC.00211-17. PMC5527630 28559256

[23] Lin Y-W, Zhou QT, Han M-L, Chen K, Onufrak NJ, Wang J, *et al*. Elucidating the pharmacokinetics/pharmacodynamics of aerosolized colistin against multidrug-resistant *Acinetobacter baumannii* and *Klebsiella pneumoniae* in a mouse lung infection model. Antimicrob Agents Chemother. 2018;62:e01790-17. 10.1128/AAC.01790-17. PMC5786800 29229637

[24] Ahmed MU, Azad MAK, Li M, Creek DJ, Han M, Zhou F, *et al*. Polymyxin-induced metabolic perturbations in human lung epithelial cells. Antimicrob Agents Chemother. 2021;65:e00835-21. 10.1128/AAC.00835-21. PMC8376244 34228550

[25] Hanafin PO, Kwa A, Zavascki AP, Sandri AM, Scheetz MH, Kubin CJ, *et al*. A population pharmacokinetic model of polymyxin B based on prospective clinical data to inform dosing in hospitalized patients. Clin Microbiol Infect. 2023;29:1174–81. 10.1016/j.cmi.2023.05.018. 37217076

[26] Liu X, Yang L, Wang M, Wang Y, Guo B, Zhang C, *et al*. Aerosolized delivery resulting in high polymyxin B concentration levels in epithelial lining fluid ensures efficacy in ventilator-associated pneumonia. JAC-Antimicrob Resist. 2025;7:dlaf023. 10.1093/jacamr/dlaf023. PMC11954587 40170884

[27] Li X, Zhou L, Wang D, Wu Q, Huang X, Zhang H, *et al*. Population pharmacokinetics study on nebulized and intravenous administration of polymyxin B in patients with pneumonia caused by multidrug-resistant gram-negative bacteria. Antimicrob Agents Chemother. 2025;69:e0004425. 10.1128/aac.00044-25. PMC12057357 40237505

[28] Gontijo AVL, Grégoire N, Lamarche I, Gobin P, Couet W, Marchand S. Biopharmaceutical characterization of nebulized antimicrobial agents in rats: 2. Colistin. Antimicrob Agents Chemother. 2014;58:3950–6. 10.1128/AAC.02819-14. PMC4068596 24798284

[29] Yapa S, Li J, Patel K, Wilson JW, Dooley MJ, George J, *et al*. Pulmonary and systemic pharmacokinetics of inhaled and intravenous colistin methanesulfonate in cystic fibrosis patients: targeting advantage of inhalational administration. Antimicrob Agents Chemother. 2014;58:2570–9. 10.1128/AAC.01705-13. PMC3993267 24550334

[30] Yapa S, Li J, Porter CJH, Nation RL, Patel K, McIntosh MP. Population pharmacokinetics of colistin methanesulfonate in rats: achieving sustained lung concentrations of colistin for targeting respiratory infections. Antimicrob Agents Chemother. 2013;57:5087–95. 10.1128/AAC.01127-13. PMC3811457 23917323

[31] Boisson M, Jacobs M, Grégoire N, Gobin P, Marchand S, Couet W, *et al*. Comparison of intrapulmonary and systemic pharmacokinetics of colistin methanesulfonate (CMS) and colistin after aerosol delivery and intravenous administration of CMS in critically ill patients. Antimicrob Agents Chemother. 2014;58:7331–9. 10.1128/AAC.03510-14. PMC4249558 25267660

[32] Wu M, Feng K, Wu X, Liu C, Zhu S, Martins FS, *et al*. Prediction of tissue exposures of polymyxin-B, amikacin and sulbactam using physiologically-based pharmacokinetic modeling. Front Microbiol. 2024. 10.3389/fmicb.2024.1435906. PMC11491386 39435440

[33] Abdelraouf K, Chang K-T, Yin T, Hu M, Tam VH. Uptake of polymyxin B into renal cells. Antimicrob Agents Chemother. 2014;58:4200–2. 10.1128/AAC.02557-14. PMC4068554 24733472

[34] Guo C, Xi L, Qu X, Huang Z, Li S, Li W, *et al*. The pharmacokinetic-nephrotoxicity relationships of CMS and CMS-E2 from the perspective of plasma and kidney drug concentrations in rats. Sci Rep. 2025;15:16299. 10.1038/s41598-025-96407-7. PMC12065821 40348891

[35] Qi B, Gijsen M, De Vocht T, Deferm N, Van Brantegem P, Abza GB, *et al*. Unravelling the hepatic elimination mechanisms of colistin. Pharm Res. 2023;40:1723–34. 10.1007/s11095-023-03536-7. 37258948

[36] Poursoleiman A, Karimi-Jafari MH, Zolmajd-Haghighi Z, Bagheri M, Haertlé T, Behbehani GR, *et al*. Polymyxins interaction to the human serum albumin: a thermodynamic and computational study. Spectrochim Acta A Mol Biomol Spectrosc. 2019;217:155–63. 10.1016/j.saa.2019.03.077. 30933779

[37] Liu J, Shao M, Xu Q, Liu F, Pan X, Wu J, *et al*. Low-dose intravenous plus inhaled versus intravenous polymyxin B for the treatment of extensive drug-resistant Gram-negative ventilator-associated pneumonia in the critical illnesses: a multi-center matched case-control study. Ann Intensive Care. 2022;12:72. 10.1186/s13613-022-01033-5. PMC9357592 35934730

[38] Zhang Y, Chen J, Fang L, Wang M, Zeng W, Ding Y. Intravenous combined with nebulized polymyxin B may be effective in treating carbapenem-resistant gram-negative bacilli hospital-acquired pneumonia: a retrospective cohort study. Eur J Clin Microbiol Infect Dis. 2025;44:2427–37. 10.1007/s10096-025-05208-3. PMC12484255 40632377

[39] Martinez MN. Factors influencing the use and interpretation of animal models in the development of parenteral drug delivery systems. AAPS J. 2011;13:632–49. 10.1208/s12248-011-9303-8. PMC3231859 21971647

[40] Wen H, Sadiq MW, Friberg LE, Svensson EM. Translational physiologically based pharmacokinetic modeling to predict human pulmonary kinetics after lung delivery. CPT Pharmacomet Syst Pharmacol. 2025;14:796–806. 10.1002/psp4.13316. PMC12001274 40087843

[41] Abdellatif S, Trifi A, Daly F, Mahjoub K, Nasri R, Ben Lakhal S. Efficacy and toxicity of aerosolised colistin in ventilator-associated pneumonia: a prospective, randomised trial. Ann Intensive Care. 2016;6:26. 10.1186/s13613-016-0127-7. PMC4816935 27033711

[42] Kim YK, Lee JH, Lee H-K, Chung BC, Yu SJ, Lee H-Y, *et al*. Efficacy of nebulized colistin-based therapy without concurrent intravenous colistin for ventilator-associated pneumonia caused by carbapenem-resistant *Acinetobacter baumannii*. J Thorac Dis. 2017;9:555–67. 10.21037/jtd.2017.02.61. PMC5394082 28449463

[43] Ahmed MU, Li J, Zhou QT. Tobramycin reduces pulmonary toxicity of polymyxin B via inhibiting the megalin-mediated drug uptake in the human lung epithelial cells. Pharmaceutics. 2024;16:389. 10.3390/pharmaceutics16030389. PMC10975719 38543283

[44] Appendix F: Estimating Human Equivalent Concentrations Using the U.S. EPA Default Approach 2008.

[45] Quigley R, Lisec A, Baum M. Ontogeny of rabbit proximal tubule urea permeability. Am J Physiol Regul Integr Comp Physiol. 2001;280:R1713–8. 10.1152/ajpregu.2001.280.6.R1713. PMC4126161 11353675

