# Supplementary material for: Physiologically Based Pharmacokinetic Modeling of Inhaled Polymyxin B: From Rabbit Optimization to Human Predictions
Source: Pharm Res. 2026 Sep 17;43(10):3525–37. doi: 10.1007/s11095-026-04194-1 (PMC13736267; doi:10.1007/s11095-026-04194-1)
Supplement: Supplementary file 1 [file 11095_2026_4194_MOESM1_ESM.docx]

**SUPPLEMENTARY MATERIALS**

**Physiologically based pharmacokinetic modeling of inhaled polymyxin B: from rabbit optimization to human predictions**

Ramya Mahadevan^1,2^*, Shekhar Yeshwante^3^*, Rajnikant Sharma^1^, Quentin Valle^1^, ChunFu Cheng^1^, Rani S. Sellers^4^, Maria Soledad Ramirez^5^, Jian Li^6^, Qi Tony Zhou^7^, David Z.D’Argenio^8^, Gauri G. Rao^1,2^

**Figure S1:** Distribution of aerosolized polymyxin B in the kidney following intratracheal (IT) administration. Kidney sections collected at (**A**) 0.5 and (**B**) 6 hours post-dose are shown. Insets display magnified views of the regions outlined by white squares in the left and right kidneys. Darker red staining indicates areas with higher polymyxin B signal or intensity or concentration, whereas lighter red staining reflects lower polymyxin B distribution.

**Figure S2**: **Visual predictive check of the PBPK model for polymyxin B PK following subcutaneous (SC) and intratracheal (IT) administration in rabbits**. Model-predicted concentration-time profiles in plasma, kidney, lung, ELF, liver, and spleen are shown for (**A**) SC and (**B**) IT administration. Solid lines represent median predicted concentrations, shaded regions represent the 95% prediction interval, and symbols represents observed data. The horizontal dashed line represents the lower limit of quantification (LLOQ; 0.055 mg/L).

**Figure S3:** **PBPK model-based simulations of polymyxin B plasma concentrations following intravenous administration in 17 subjects**. Individual subject dosing information was used for the simulations. Solid line represents model simulated concentrations, and dots represent observed data. Observed data for Subjects 1 and 2 correspond to mean concentrations from healthy volunteers (Liu et al, 2021), whereas data for Subjects 3-17 were obtained from critically ill patients (Subjects 3 - 13: Hanafin et al 2023; Subjects 14 – 17: Matcha et al., unpublished data).

**Figure S4: Goodness-of-fit plots comparing simulated versus observed pharmacokinetic metrics (AUC) for polymyxin B administered intravenously in critically ill patients with and without AKI (n=117).** The solid line represents the line of identity, while the dotted and dashed lines indicate 1.25-fold and 2-fold deviation boundaries, respectively.**Figure S5:** Receiver operating characteristic **(**ROC) curves evaluating the sensitivity and specificity of PK parameters for predicting polymyxin B-induced acute kidney injury (AKI).

**AUC_SS,24h,kidneyev_** : area under the steady-state concentration-time curve in the kidney extravascular region; **AUC_SS,24h,plasma_** : area under the total steady state concentration-time curve in plasma; **fAUC_SS,24h,plasma_** : area under the unbound steady state concentration-time curve in plasma; **fAUC_0-24h,plasma_** : area under the unbound concentration curve-time (0 to 24 h) in plasma; **C_min,SS,kidney_** : trough concentration at steady state in the kidney extravascular region; **C_min,SS,plasma_** : trough concentration at steady state in plasma; **fC_min,SS,plasma_** : trough unbound concentration at steady state in plasma; **fC_min,plasma_** : trough concentration observed within first 24 h of treatment in plasma; **C_max,SS,plasma_** : peak concentration at steady state in plasma; **fC_max,SS,plasma_** : peak unbound concentration at steady state in plasma; **fC_max,plasma_** : peak unbound concentration from 0 to 24 h in plasma.

**Figure S6:** Probability of PK/PD target attainment (PTA) in plasma and epithelial lining fluid (ELF) and probability of toxicity attainment (PToXA) in plasma, kidney, and ELF following (**A**) inhalation (INH) monotherapy, (**B**) intravenous (IV) monotherapy, (**C**) combined IV + INH therapy against *Klebsiella pneumoniae.*

**Figure S7:** Probability of PK/PD target attainment (PTA) in plasma and epithelial lining fluid (ELF) and probability of toxicity attainment (PToXA) in plasma, kidney, and ELF following (**A**) inhalation (INH) monotherapy, (**B**) intravenous (IV) monotherapy, (**C**) combined IV + INH therapy against *Pseudomonas aeruginosa.*

**Figure S8:** The mechanism-based PK/PD model schematic for bacterial killing by polymyxin B (PMBkill) as monotherapy. Bacterial replication was modeled using the two-state bacterial life cycle model, in which each bacterial subpopulation was divided into a vegetative (state 1) and replicating state (state 2). K_12_ is a first-order growth constant that controls the transition of bacteria in state 1 to state 2 for each proposed bacterial subpopulation, and K_21_ is a rapid first-order process that controls the doubling process. The model consisted of two subpopulation that is susceptible (PMB_S_) and resistant (PMB_R_) to polymyxin B. All model parameters are described in **Table S6**.

**Figure S9:** Mechanism based PK/PD model predicted bacterial counts for treatment with polymyxin B monotherapy across two *A. baumannii* isolates ATCC19606 (A) and Ab124 (B). The solid lines represent model predictions, and the symbols denote observed data.

**Table S1:** Rabbit physiological model parameters.

| **Parameter** | **Value** | **Reference** |
| --- | --- | --- |
| Hematocrit | 0.36 | [1] |
| Weight (kg) | 3.2 |  |
| Cardiac output (L/h) | 38.185 | [1] |
| V_ELF_ (L) | 0.00122 | [2] |
| GFR (L/h) | 0.62 | [3] |
| **Tissue volumes - Fraction of body weight** | | |
| FV_artery_ | 0.02 | [1,4] |
| FV_venous_ | 0.048 |  |
| FV_lung_ | 0.0068 |  |
| FV_brain_ | 0.0056 |  |
| FV_heart_ | 0.0024 |  |
| FV_muscle_ | 0.54 |  |
| FV_skin_ | 0.044 |  |
| FV_adipose_ | 0.0375 |  |
| FV_liver_ | 0.0287 |  |
| FV_GI_ | 0.027 |  |
| FV_kidney_ | 0.0052 |  |
| FV_kidney,vascular_ | 0.142 |  |
| FV_spleen_ | 0.0004 |  |
| **Tissue Blood flow - Fraction of cardiac output** | | |
| FQ_brain_ | 0.018 | [1,4] |
| FQ_heart_ | 0.036 |  |
| FQ_skin_ | 0.077 |  |
| FQ_muscle_ | 0.293 |  |
| FQ_adipose_ | 0.060 |  |
| FQ_GI_ | 0.131 |  |
| FQ_spleen_ | 0.017 |  |
| FQ_hepatic artery_ | 0.07 |  |
| FQ_kidney_ | 0.174 |  |
| **Others** | | |
| FV_kidney_tubular_ (Fraction of kidney volume) | 0.2 | [5] |
| Urine flow (mL/day) | 150 | [1] |

**Table S2:** Polymyxin B drug specific parameters

| **Parameter** | **Value** | **Reference** |
| --- | --- | --- |
| Molecular weight | 1203.5 g/mol |  |
| Compound type | Ampholyte |  |
| *logP* | -4.5 | [6] |
| *pKa* | 9.07, 9.54,9.84,10.02,10.24 | [7] |
| Fraction unbound in plasma (*fup*) | 0.42 | [8] |
| Fraction excreted in urine | 0.04 | [8] |
| Blood-to-plasma ratio | 0.64 | 1-Hematocrit |
| *Kp_lung_* | 0.141 | [9] |
| *Kp_spleen_* | 0.087 |  |
| *Kp_liver_* | 0.068 |  |
| *Kp_brain_* | 0.068 |  |
| *Kp_heart_* | 0.134 |  |
| *Kp_skin_* | 0.160 |  |
| *Kp_muscle_* | 0.050 |  |
| *Kp_adipose_* | 0.057 |  |
| *Kp_GI_* | 0.118 |  |

**Table S3:** Published studies used for validating human PBPK model simulations

| **Publication** | **Subjects Type/ Demographics^a^** | **Dosage Regimen** | **Sample** | **Model Simulation^b^** |
| --- | --- | --- | --- | --- |
| Liu et al., 2021 [10] | Healthy (n=10)  Age:19ꟷ45 years  Bodyweight: 59.5 – 113.6 kg | 0.75 mg/kg (1h inf)  1.5 mg/kg (1.5 h inf) | Plasma | 10 subjects were simulated with 0.75 mg/kg dosage regimen.    10 subjects were simulated with 1.5 mg/kg dosage regimen.  *CLp* was allometrically scaled to humans. |
| Hanafin et al., 2023 [11] | Critically ill (n=11)  Age: 50ꟷ86 years  Bodyweight: 57ꟷ130 kg  CrCl: 13.59ꟷ65.22 ml/min  fup: 0.26ꟷ0.54 | 1 – 2.3 mg/kg q12h  (1 – 6 h inf) | Plasma | 11 representative subjects were simulated using their individual dosing and demographics (age, bodyweight, sex, fup, and CrCl) information.  *CLp* was allometrically scaled to humans. |
| Matcha et al., 2026 [12] | Critically ill (n=4)  Age: 69ꟷ90 years  Bodyweight: 40ꟷ75 kg  CrCl: 21.61-94.03 ml/min | 0.67 – 2.5 mg/kg q12h  (1 h inf) | Plasma | 4 representative subjects were simulated with their individual dosing and demographics (age, bodyweight, sex, fup, and CrCl) information.  *CLp* was allometrically scaled to humans. |
| Liu et al., 2025^c^ [13] | Ventilator-associated pneumonia patients (n=4)  Age: 16-67 years  Body weight: 51-77.5 kg | IV: 50 – 100 mg q12h (1h inf)  Aerosolized: 25 – 50 mg q12h (NebuEz mesh nebulizer for 30 min) | Plasma and ELF | 4 subjects receiving IV and aerosolized regimen simultaneously were simulated with their individual dosing and demographics (age, body weight) information.  As vibrating mesh nebulizer was used, which has higher drug deposition, fraction of drug available in ELF after nebulization was assumed to be 0.9.  *S_Kpu,lun,IT_* was optimized to 0.25 and *Cl_ELF_* was optimized to 0.001 L/h to explain the observed ELF data.  *CLp* and *Cl_ELF_* were allometrically scaled to humans. |
| Li et al., 2025^c^ [14] | Pneumonia patients  (n=76)    Age: 55-76.3 years  Body weight: 54.38-70 kg  CrCL: 35.4 – 101.3 ml/min | IV loading dose: 100 – 150 mg (1h inf)  Maintenance dose: 40 – 75 mg q12h (1h inf)  Nebulized: 25 mg q12h via vibration mesh nebulizer or jet nebulizer over 15 mins. | Plasma and ELF | As this is population data, we simulated 1000 virtual subjects based on the demographics provided. Subjects received IV + nebulized regimen. Simulations incorporated 30% variability on *CLp_human_, PS_diff_, CL_reab_, Kbind, Kunbind, Kp_rem_, S_Kp,lun_, S_Kpu,lun_, S_Kp,lun,IT_, S_Kpu,lun,IT_ PSlung* and *CL_ELF,human_* and 30% residual unexplained variability  Fraction available in ELF after nebulization was reported to be 0.51 to 0.60.  *S_Kpu,lun,IT_* was optimized to 0.25 and *Cl_ELF_* was optimized to 0.001 L/h to explain the observed ELF data.  *CLp* and *Cl_ELF_* were allometrically scaled to humans. |

^a^Missing demographics were calculated as shown in Table S4. Where fup was not reported, 0.42 was assumed for individual simulations and 0.14-0.61 for population simulations.

^b^Physiological parameters were derived from study-specific demographics (**Table S4**)

^c^Digitized using Webplotdigitizer digitized from published studies using WebPlotDigitizer (v4.6)

**Table S4:** Human physiological model parameters

| **Parameter** | **Equation / Value** | **Reference** |
| --- | --- | --- |
| Hematocrit | Male: 0.43, CV (%):6.5 | [15] |
|  | Female: 0.38, CV (%): 7.1 |  |
| Height | Male: $175.32+0.1113*age-0.0025* age^{2}$  CV (%): 3.9 |  |
|  | Female: $161.66 + 0.1319 * age - 0.0027 *age^{2}$  CV (%): 3.9 |  |
| Weight | Male:$exp(2.643 + 0.0099 * Height)$  CV (%): 15 |  |
|  | Female: $exp (2.7383 + 0.0091 * Height)$  CV (%): 18.8 |  |
| BSA | Male: $0.007184*Height^{0.725}*Weight^{0.425}$ |  |
|  | Female: $0.007184*Height^{0.725}*Weight^{0.425}$ |  |
| **Volumes** **(L)** | | |
| V_lung_ | Male: $((29.08 * (Height/100) * Weight^0.5 + 11.06 + 35.47 * (Height/100) * Weight^0.5 + 5.53)/1000)/1.05$ | [15] |
|  | Female: $((31.46 * (Height/100) * Weight^0.5 + 1.43 + 35.3 * (Height/100) * Weight^0.5 +$*1.53)/1000)/1.05* |  |
| V_adipose_ | Male: $1.36*Weight)/(Height/100)-42$ |  |
|  | Female: $1.61*Weight)/(Height/100)-38.3$ |  |
| V_bone_ | Male: $((Weight-Vadipose*0.92)/1.3*0.058$ |  |
|  | Female: $((Weight-Vadipose*0.92)/1.3*0.051$ |  |
| V_heart_ | Male: $((155.18*\left( BSA \right)^{1.29})/1000)/1.05$ |  |
|  | Female: $((124.13*\left( BSA \right)^{1.242})/1000)/1.05$ |  |
| V_kidney_ | $((15.4+2.04*Weight+51.8*(Height/100^2)/1000)/1.05$ |  |
| V_kidney,vascular_ | $0.054176*Vkidney$ | [16] |
| V_kidney,tubular_ | $0.2*Vkidney$ | [5] |
| V_kidney,extravascular_ | $Vkidney-(Vkidney_{vascular}+Vkidney_{tubular})$ |  |
| V_muscle_ | Male: $(0.244*Weight+7.8*(Height/100)-0.098*age+3.3)/1.04$ | [15] |
|  | Female: $(0.244*Weight+7.8*(Height/100)-0.098*age-3.3)/1.04$ |  |
| V_skin_ | $(BSA/1000)*45.655+(BSA/1000) *1240$ |  |
| V_spleen_ | $(6.516*Weight^{0.797})/1000$ |  |
| V_artery_ | Male:$(((13.1*Height+18.05*Weight-480)/0.5723)/1000)/2$ |  |
|  | Female: $(((35.5*Height+2.27*Weight-3382)/0.6178)/1000)/2$ |  |
| V_venous_ | Male: $(((13.1*Height+18.05*Weight-480)/0.5723)/1000)/2$ |  |
|  | Female: $(((35.5*Height+2.27*Weight-3382)/0.6178)/1000)/2$ |  |
| V_GI_ | $(0.021*\left( Weight-Vadipose*0.92 \right))/1.05$ |  |
| V_brain_ | $(1.449-3.62/Weight)/1.04$ |  |
| V_liver_ | $(\left( 1072.8*\left( BSA \right) \right)-345.7)/1000$ |  |
| V_ELF_ | 0.025; CV(%):20 | [17] |
| V_rest_ | $Weight-V_{venous}-V_{artery}-V_{liver}-V_{GI}-V_{spleen}-V_{skin}-V_{muscle}-V_{kidney}-V_{heart}-V_{adipose}-V_{lung}-V_{brain}$ |  |
| **Blood flow (L/h)** | | |
| Q_co_ | $BSA*60*(3-0.01*\left( age-20 \right))$ | [15] |
| Q_adipose_ | Male: $Qco*0.05$ |  |
|  | Female: $Qco*0.085$ |  |
| Q_heart_ | Male: $Qco*0.04$ |  |
|  | Female: $Qco*0.05$ |  |
| Q_kidney_ | Male: $Qco*0.19$ |  |
|  | Female: $Qco*0.17$ |  |
| Q_muscle_ | Male: $Qco*0.17$ |  |
|  | Female: $Qco*0.12$ |  |
| Q_skin_ | $Qco*0.05$ |  |
| Q_spleen_ | Male: $Qco*0.02$ |  |
|  | Female: $Qco*0.03$ |  |
| Q_GI_ | Male: $Qco*0.15$ |  |
|  | Female: $Qco*0.17$ |  |
| Q_liver_ | Male: $Qco*0.235$ |  |
|  | Female: $Qco*0.265$ |  |
| Q_brain_ | $Qco*0.12$ |  |
| **Others** | | |
| Urine flow (mL/day) | 1400 | [1] |
| GFR (ml/min) | 125 |  |
| Surface area of lung (*SA_lung_*) (Cm^2^) | 543400 | [18] |
| Surface area of kidney proximal tubular cells (*SA_kidney,PTC_*) (Cm^2^) | 610700 | [19] |

**Table S5: Non-compartmental analysis (NCA) of observed plasma pharmacokinetic parameters following subcutaneous and intratracheal administration of polymyxin B in rabbits ^a^.**

| **PK parameter** | **Plasma** | **Lung** | **ELF** | **Kidney** | **Liver** | **Spleen^c^** |
| --- | --- | --- | --- | --- | --- | --- |
| **Subcutaneous Polymyxin B** | | | | | | |
| Cmax (mg/L) | 10.4 | 0.41 | 0.64 | 11 | 0.68 | 0.81 |
| Tmax (h) | 0.33 | 0.5 | 6 | 2 | 0.5 | 2 |
| AUC (mg*h/L) | 27.6 | 1.21 | 2.87 | 50.7 | 1.39 | 3.31 |
| T1/2 (h)^b^ | 2.22 | 3.78 | - | 10 | 1.78 | 3.49 |
| **Intratracheal polymyxin B** | | | | | | |
| Cmax (mg/L) | 5.33 | 6.3 | 18.1 | 3.61 | 3 | - |
| Tmax (h) | 0.5 | 0.5 | 0.5 | 2 | 2 | - |
| AUC (mg*h/L) | 14.6 | 15.8 | 67.4 | 18.2 | 6.88 | - |
| T1/2 (h)^b^ | 1.68 | - | 2.88 | - | 1.13 | - |

Cmax: maximum observed plasma concentration; Tmax: time to maximum observed plasma concentration; AUC: area under the plasma concentration–time curve; T1/2: terminal elimination half-life

^a^ NCA was performed using the mean concentration–time data.

^b^ T1/2 was not calculated because of insufficient data to characterize the terminal elimination phase.

^c^ NCA parameters were not calculated because only one concentration–time observation was available for the spleen following intratracheal administration.

**Table S6:** Comparison of PK parameters in AKI vs non-AKI group^a^

| **Parameter^b^** | **AKI (n=62)^c^**  **Median (IQR)** | **non-AKI (n=55)^c^**  **Median (IQR)** | **p-value** |
| --- | --- | --- | --- |
| AUC_0-24h,plasma_ | 89.2 (79-103) | 82.7 (70.6-103) | 0.197 |
| C_min,plasma_ | 1.51 (1.23-1.97) | 1.49 (1.15-1.69) | 0.072 |
| C_max,plasma_ | 7.99 (6.96-8.99) | 7.75 (6.42-10.3) | 0.878 |
| AUC_SS,24h,plasma_ | 97.5 (87.8-111) | 88.2 (80.7-99.2) | 0.003 |
| C_min,SS,plasma_ | 1.59 (1.27-1.98) | 1.52 (1.18-1.73) | 0.049 |
| C_max,SS,plasma_ | 8.16 (7.13-8.97) | 7.35 (6.53-8.45) | 0.038 |
| fAUC_0-24h,plasma_ | 33.9 (25.1-46.1) | 26.9 (19.1-36.9) | 0.001 |
| fC_min,plasma_ | 0.63 (0.38-0.88) | 0.41 (0.26-0.64) | 0.000 |
| fC_max,plasma_ | 3.02 (2.22-3.95) | 2.53 (1.8-3.39) | 0.024 |
| fAUC_SS,24h,plasma_ | 36.7 (29.7-48.9) | 27.5 (20.4-34.6) | 0.000 |
| fC_min,SS,plasma_ | 0.64 (0.4-0.9) | 0.42 (0.29-0.64) | 0.001 |
| fC_max,SS,plasma_ | 3.03 (2.4-3.65) | 2.33 (1.81-2.91) | 0.000 |
| AUC_0-24h,kidney_ | 256 (221-313) | 249 (197-351) | 0.619 |
| C_min,kidney_ | 11.2 (9.81-13.6) | 10.4 (8.35-14.2) | 0.249 |
| C_max,kidney_ | 17.1 (14.6-21.2) | 16.3 (13.8-22.4) | 0.646 |
| AUC_0-24h,kidneyev_ | 147 (130-178) | 140 (114-196) | 0.441 |
| C_min,kidneyev_ | 2.83 (2.12-3.49) | 2.45 (2-3.11) | 0.105 |
| C_max,kidneyev_ | 12.4 (10.3-14.6) | 12.3 (9.54-17.7) | 0.913 |
| AUC_SS,24h,kidney_ | 661 (590-800) | 607 (509-724) | 0.057 |
| C_min,SS,kidney_ | 25.4 (22.2-29.6) | 22.8 (18.7-27.0) | 0.045 |
| C_max,SS,kidney_ | 33.6 (30.3-40.8) | 30.4 (25.9-37.1) | 0.090 |
| AUC_SS,24h,kidneyev_ | 171 (149-197) | 153 (126-179) | 0.023 |
| C_min,SS,kidneyev_ | 3.01 (2.33-3.6) | 2.59 (2.08-3.2) | 0.072 |
| C_max,SS,kidneyev_ | 13.1 (10.8-15.0) | 12.2 (9.75-14.4) | 0.144 |

^a^ using Mann-Whitney U tests

^b^ AUC_0-24h,plasma_ : area under the total concentration curve from 0 to 24 h in plasma

C_min,plasma_ : trough concentration observed within first 24 h of treatment in plasma;

C_max,plasma_ : peak concentration observed within first 24 h of treatment in plasma;

AUC_SS,24h,plasma_ : area under the total steady-state concentration curve in plasma;

C_min,SS,plasma_ : trough concentration at steady state in plasma;

C_max,SS,plasma_ : peak concentration at steady state in plasma;

fAUC_0-24h,plasma_ : area under the unbound concentration curve from 0 to 24 h in plasma;

fC_min,plasma_ : trough unbound concentration within first 24 h of treatment in plasma;

fC_max,plasma_ : peak unbound concentration within first 24 h of treatment in plasma;

fAUC_SS,24h,plasma_ : area under the unbound steady-state concentration curve in plasma;

fC_min,SS,plasma_ : trough unbound concentration at steady state in plasma;

fC_max,SS,plasma_ : peak unbound concentration at steady state in plasma;

AUC_0-24h,kidney_ : area under the concentration curve from 0 to 24 h in total kidney;

C_min,kidney_ : trough concentration observed within first 24 h of treatment in total kidney;

C_max,kidney_ : peak concentration observed within first 24 h of treatment in total kidney;

AUC_0-24h,kidneyev_ : area under the concentration-time curve from 0 to 24 h in the extravascular region of kidney;

C_min,kidneyev_ : trough concentration observed within first 24 h of treatment in extravascular region of kidney;

C_max,kidneyev_ : peak concentration observed within first 24 h of treatment in extravascular region of kidney;

AUC_SS,24h,kidney_ : area under the steady-state concentration-time curve in total kidney;

C_min,SS,kidney_ : trough concentration at steady state in total kidney;

C_min,SS,kidney_ : trough concentration at steady state in total kidney;

AUC_SS,24h,kidneyev_ : area under the steady-state concentration-time curve in the extravascular region of kidney;

C_min,SS,kidney_ : trough concentration at steady state in the extravascular region of kidney;

C_min,SS,kidney_ : trough concentration at steady state in the extravascular region of kidney;

^c^Steady-state PK parameters were compared between AKI (n=58) and non-AKI (n=50) cohorts, excluding nine subjects who did not receive dosing up to steady state.

**Table S7:** Final parameter estimates for the mechanism-based PK/PD model describing polymyxin B activity

| **Parameter Estimate (%CV)** | **GR936** | **GR1010** |
| --- | --- | --- |
| **Bacterial growth and subpopulations** |  |  |
| Bacterial initial inoculum (LogCFU0) [log_10_ CFU/mL] | 6.39 (1.52) | 6.69 (5.09) |
| Maximum population size (LogCFUmax) [log_10_ CFU/mL] | 8.49 (1.16) | 8.34 (2.26) |
| Replication rate constant (k21) [h^-1^] | 50 (fixed) | 50 (fixed) |
| Mean generation time (MGT) [min] |  |  |
| PMB^S^ | 97.7 (12.1) | 52.8 (7.53) |
| PMB^R^ | 34.9 (8.69) | 38.3 (10.3) |
| Log mutant frequency at baseline (log_10_ MF) |  |  |
| PMB^R^ | -6.80 (1.56) | -2.38 (4.79) |
| **Drug effect of PMB** |  |  |
| Linear function for PMB drug effect on S subpopulation (K_slope,PMB,S_) [mg/L] | 5.48 (8.35) | 9.93 (16.9) |
| Linear function for PMB drug effect on S subpopulation (K_slope,PMB,R_) [mg/L] | 0.13 (6.78) | 0.47 (6.01) |
| Hill coefficient of PMB | 0.68 (5.58) | 0.71 (5.29) |
| **Variability model** |  |  |
| Additive residual variability [log_10_ CFU/mL] | 0.32 (17.8) | 0.24 (19.2) |

**METHODS:**

**Chemicals**

Clinical grade PMB (AuroMedics Pharma LLC, East Windsor, NJ), ketamine hydrochloride (KetaVed^®^, Vedco Inc., Sait Joseph, MO), dexmedetomidine hydrochloride (Dexmedesed^TM^, Dechra Veterinary Products, Northwich, UK), atipamezole hydrochloride (Revertidine^TM^, Modern veterinary therapeutics LLC, Coral Gables, FL) were purchased from their respective manufacturers. Pentobarbital sodium (Euthasol^®^) for euthanasia was procured from Virbac Corporation (Westlake, TX). Anti-PMB mouse IgM antibody (clone 45) was obtained from Invitrogen (Waltham, MA), and goat anti-mouse IgM conjugated with Alexa Fluor 647 was purchased from Thermo Fisher Scientific (Waltham, MA).

**Endotracheal intubation and monitoring**

Once unresponsive to toe pinch after anesthesia, a 3 mm inner diameter (4.2 mm outer diameter) endotracheal tube (ET, VET one^®^, Boise, ID) was inserted into the trachea and advanced until a continuous whistle sound confirmed correct placement. After securing the ET tube, rabbits were connected to an anesthesia circuit, and ventilation was verified by monitoring respiratory rate (breaths per minute) and skin color. The flexible aerosolizer needle was premarked based on the ET tube to ensure insertion into the tracheal lumen without contacting lung tissue. After aerosolization, the needle was carefully withdrawn, and the rabbit was placed in the sternal position with the head slightly extended to facilitate drug distribution. All collected blood samples were stored on ice until centrifugation for plasma separation.

**Quantification of polymyxin B**

Polymyxin B1 and B2 were quantified in plasma and tissue using a modified LC-MS/MS method [20]. Analyses were performed on an API 4000 triple quadrupole mass spectrometer with a turbo ion spray source and an Agilent 1100 HPLC system (Santa Clara, CA) equipped with a Waters (Milford, MA) Xterra C18 column (3.5 μm x 2.1 mm x 100 mm) and Vanguard Cartridge (2.1 mm x 5 mm). Tissue samples were homogenized in 50/50 (v/v) ethanol/deionized water using a Geno/Grinder^®^ and homogenization beads with solvent added at a 5:1 ratio (solvent:tissue weight). Polymyxin B1, B2, and E2 standards were prepared in ultra-pure water and diluted with sample diluent (5/95 v/v acetonitrile/deionized water + 0.1% formic acid). Intermediate stock solutions were spiked (50 μL) into 450 μL of rat plasma to prepare calibration standards, which underwent the same extraction procedure as rabbit samples. Polymyxin E2 (colistin B) served as the internal standard. Calibration standards were freshly prepared in rat plasma for each analysis. Calibration curves (5 ng/mL to 4000 ng/mL) were generated in rat plasma, achieving LOQs of 5 ng/mL for polymyxin B1 and 50 ng/mL for polymyxin B2. Samples (10 μL) were injected, with the autosampler and column maintained at 10°C and 30°C, respectively. Elution was performed at 0.3 mL/min using mobile phases A (deionized water containing 0.5% formic acid [FA] and 0.01% trifluoroacetic acid [TFA]) and B (50:50 [v/v] acetonitrile [ACN]/methanol [MeOH] containing 0.5% FA and 0.01% TFA) with the following gradient: 82% A (0–4.5 min), 70% A (4.5–7.5 min), 60% A (7.5–13 min), 5% A (14 min), and re-equilibration to 82% A (14.5–21 min); total run time 21 min.

**Quantification of polymyxin B in ELF**

Urea concentrations in plasma (𝑈𝑟𝑒𝑎_𝑃𝑙𝑎𝑠𝑚𝑎_) and BALF (𝑈𝑟𝑒𝑎_𝐵𝐴𝐿𝐹_) were measured using the QuantiChrom™ Urea Assay Kit (detection limit 13 μM; BioAssay Systems, Hayward, CA). ELF volume (𝑉_𝐸𝐿𝐹_) was calculated using Equation (1) based on urea concentrations in plasma and BALF. PMB concentration in ELF (𝐶_𝐸𝐿𝐹_) was then derived from PMB concentration in BALF (𝐶_𝐵𝐴𝐿𝐹_) and 𝑉_𝐸𝐿𝐹_ using Equation (2) [21]

$$V_{ELF}=V_{BALF}\cdot\left( \frac{Urea_{BALF}}{Urea_{plasma}} \right) (1)$$

$$C_{ELF}=C_{BALF}\cdot\left( \frac{V_{BALF}}{V_{ELF}} \right) (2)$$

**Calculating the unbound fraction in the ELF**

The unbound fraction in the ELF was calculated using Equation (3). Consistent with previous studies, the protein composition of ELF was assumed to be similar to plasma, with an ELF-to-plasma protein concentration ratio of 0.6 [22,23]:

$fu_{ELF}=\frac{fu_{p}}{0.6+0.4\cdot fu_{p}}$	(Eq. 3)

where *fup* is the unbound fraction in plasma.

**MECHANISM-BASED PK/PD MODEL**

**Antibiotics, medium, and bacterial isolates**

Two *Acinetobacter baumannii* strains, ATCC19606 and Ab124 (clinical isolate from Dr Ramirez) were used in this study. Cation-adjusted Mueller–Hinton broth (CAMHB: 25 µg/mL Ca^2+^, 12.5 µg/mL Mg^2+^) and Mueller–Hinton II agar (BD Biosciences, Franklin Lakes, NJ) were used for all *in vitro* models. The antibiotics polymyxin B (Sigma-Aldrich, St. Louis, MO) were freshly prepared in sterile phosphate-buffered saline and filter sterilized using a 0.22 μm Millex GP syringe filter (Corning Inc., Corning, NY).

**Static concentration time-kill (SCTK) assays**

SCTK assays were conducted over 24 hours to characterize the rate and extent of killing by polymyxin B against two *A .baumannii* strains (ATCC19606 and Ab124), as previously described [24]. Polymyxin B was evaluated at concentrations of 1, 16, 64, 128, and 256 mg/L. These concentrations were selected to span a broad range of free drug exposures encompassing clinically achievable steady state plasma concentrations following intravenous administration (Cmax_SS_: 2 – 14 mg/L) as well as ELF concentrations reported after inhaled delivery (20.6 ꟷ 97.6 mg/L) [11,25]. Samples were collected at 0, 1, 2, 4, 6, 8, and 24 hours for quantification of viable bacteria. At each time point, 1 mL samples were obtained, serially diluted in PBS, and 50 µL diluted sample was plated on Muller-Hinton agar (MHA) plates using an automated spiral plater (easySpiral, Interscience, Saint Nom la Breteche, France). Plates were incubated at 37°C for 18–24 hours, and viable bacterial colonies were counted using a ProtoCOL automated colony counter (Symbiosis, Cambridge, UK). The lower limit of quantification is 1.3 log_10_ CFU/mL.

**Model development**

A mechanism-based PK/PD model was developed using SCTK time course data to characterize bacterial burden dynamics over time in response to polymyxin treatment. A mixture model with pre-existing bacterial subpopulations of differing susceptibilities [i.e., susceptible (S) and resistant (R)] to PMB was considered to account for the heterogeneity of the bacterial inoculum (**Figure S8**). Bacterial replication was modeled using life cycle growth model, where each subpopulation was represented in two states: vegetative (state 1) and replicating (state 2) [24]. The total bacterial population is defined as:

${CFU}_{tot}={CFU}_{S,1}+{CFU}_{S,2}+{CFU}_{R,1}+{CFU}_{R,2}$	(Eq. 4)

where CFU_tot_ is the total viable bacterial concentration, and CFU_S_ and CFU_R_ represent PMB-susceptible and PMB-resistant subpopulations, each comprising vegetative (state 1) and replicative (state 2) states. The resistant subpopulation dynamics are described by Equations (5) and (6).

$\frac{d\left( {CFU}_{R,1} \right)}{dt}=REP\cdot k_{21}\cdot{CFU}_{R,2}-k_{12,R}\cdot{CFU}_{R,1}-Kill_{PMB,R}\cdot CFU_{R,1} ; ( IC_{CFU_{R,1}}= CFU_{0}\cdot MF_{R})$	(Eq. 5)

$\frac{d\left( {CFU}_{R,2} \right)}{dt}=-k_{21}\cdot{CFU}_{R,2}+k_{12,R}\cdot{CFU}_{R,1}-Kill_{PMB,R}\cdot{CFU}_{R,2} , (IC_{CFU_{R,2}}=0)$	(Eq. 6)

CFU_R,1_ and CFU_R,2_ represent the replicative and vegetative states, respectively, for each bacterial subpopulation. The transition between these states is governed by *k*_12_, the rate at which replicative bacteria become dormant, and *k_21_*, the rapid transition rate from dormancy to active division, fixed to 50 h^−1^. REP is the replication factor defined as $2\cdot\left( 1-\frac{{CFU}_{tot}}{{CFU}_{MAX}+{CFU}_{tot}} \right)$ , *CFU_MAX_* is the maximum bacterial population size, and 2 represents the doubling of bacteria during replication. The killing effect of polymyxin B (*Kill_PMB_*) was modeled using a slope function:

${Kill}_{drug}=Slope_{drug}\cdot\left( C_{drug} \right)^{h}$	(Eq. 7)

where C_drug_ is the PMB concentration, Slope_drug_ is the linear function for PMB killing effect, and h is the Hill coefficient. Initial total bacterial load (log_10_ CFU_0_) and log_10_-transformed mutation frequencies for each subpopulation were estimated. Initial conditions were implemented similar to previously published methods [24]. Final model fits and parameter estimates are shown in **Figure S9** and **Table S7** respectively.

Residual unexplained variability for log_10_-transformed bacterial counts was modeled using an additive error model. Observations below the lower limit of quantification (LLOQ) were handled using the Beal 3 method [24]. Parameters were estimated using the importance sampling algorithm (pmethod=4) in S-ADAPT v1.57 [26] facilitated by SADAPT-TRAN [27]. Model performance was assessed based on model fits, parameter plausibility and precision of parameter estimates, and the objective function value (OFV).

**RABBIT PBPK NONMEM MODEL CODE**

$PROBLEM PBPK

;; 2. Description: PMB PBPK

;; x1. Author: rmahadev

$INPUT C ID CID SEX BWT DOSE DOSE_UG AMT CMT EVID MDV TIME DV DVID

$DATA sc_dataset_upd.csv IGNORE=C

$SUBROUTINE ADVAN13 TOL=6

$MODEL

NCOMP=18

COMP=(AVEN) ; 1 venous blood (PMB)

COMP=(AART) ; 2 arterial blood (PMB)

COMP=(ABRA) ; 3 brain (PMB)

COMP=(AHRT) ; 4 heart (PMB)

COMP=(AMUS) ; 5 muscle (PMB)

COMP=(ASKN) ; 6 skin (PMB)

COMP=(AADI) ; 7 adipose (PMB)

COMP=(AGI) ; 8 GI (PMB)

COMP=(ALUN) ; 9 lung (PMB)

COMP=(ALIV) ; 10 liver (PMB)

COMP=(ASPL) ; 11 spleen (PMB)

COMP=(AKIDVAS) ; 12 kidney vascular(PMB)

COMP=(ATUB) ; 13 Kidney tubule (PMB)

COMP=(AKIDCEL) ; 14 kidney extravascular compartment (PMB)

COMP=(AKIDDEEP) ; 15 binding compartment

COMP=(ARES) ; 16 rest of body (PMB)

COMP=(DEPSC) ; 17 SC depot compartment (PMB)

COMP=(AELF) ; 18 ELF

$PK

;; --------------- Blood flows (L/H) --------------------- ;;

WT=3.2

DEB = 15.96 ; cardiac output (L/h/kg^0.75)

HT = 0.36	; hematocrit

CO = DEB*(WT**0.75)*(1-HT) ; Total plasmatic flow (L/h)

GFR = 0.62 ; Glomerular filtration rate (L/h)

QBRA = 0.018*CO ;; Brain plasmatic flow (L/h)

QHRT = 0.036*CO ;; Heart plasmatic flow (L/h)

QSKN = 0.077*CO ; ; Skin plasmatic flow (L/h)

QMUS = 0.293*CO ;; Muscle plasmatic flow(L/h)

QADI = 0.060*CO ;; Adipose plasmatic flow (L/h)

QGI = 0.131*CO ;; GIO plasmatic flow (L/h)

QSPL = 0.017*CO ;; SPL plasmatic flow (L/h)

QHEPART = 0.07*CO ;; Hepatic arterial plasmatic flow (L/h)

QLIV= QHEPART +QGI+ QSPL ;; Total hepatic plasmatic flow (L/h)

QKID = 0.174*CO ;; Kidney plasmatic flow (L/h)

QTUB = 0.67*GFR ;; Tubular flow in kidney (L/h)

SUMQ = QMUS + QKID + QHRT + QLIV + QBRA + QSKN + QADI

QRES = CO - SUMQ	;rest of body plasmatic flow (L/h)

QART = CO ;; Artery plasmatic flow

QLUN = CO ;; Lung plasmatic flow

QVEN = CO ; Vein plasmatic flow

;; ------------ Tissue volumes (L) --------------- ;;

VART = (0.02) *WT ;; Arterial volume (L);

VVEN = (0.048) * WT ;; Venous volume (L);

VLUN = (0.0068) * WT; Lung volume (L);

VBRA = (0.0056)* WT; Brain volume (L);

VHRT = (0.0024)* WT; Heart volume (L);

VMUS = (0.54)*WT ;; Muscle volume (L)

VSKN = (0.044)*WT ;; Skin volume (L);

VADI = (0.0375) *WT ;; Adipose volume (L);

VLIV = (0.0287)* WT; Liver volume (L);

VGI = (0.027)*WT ;; GIO volume stomach+Small Intestine+colon (L);

VSPL = (0.0004)*WT ;;Spleen

VKID = (0.0052) * WT ; Kidney volume (L);

VKIDVAS = (0.142)*VKID; Vascular fraction of Kidney

VTUB = (0.2)*VKID ; Tubulear fraction of Kidney

VKIDCEL = VKID- (VKIDVAS+VTUB) ; Volume of Extravascular Kidney

VRES = WT - (VART + VVEN + VLUN + VBRA + VHRT + VSKN + VMUS + VADI + VGI + VLIV + VKID + VSPL) ;; Rest of body volume

VTOT = WT; Total Volume

VBLA = 0.01

VELF = 0.00122

;; ------------------------------- Drug related Parameters -------------------------------- ;;

TVKPREM = THETA(1)

TVPAPPKID = 10**(-THETA(2))

TVSCALAR = THETA(3)

TVKUSCALAR = THETA(4)

KASC = THETA(5)	;Absorption rate constant after SC

CLP = THETA(6)	; Plasma clearance

CLREAB = THETA(7) ; Tubular reabsorption clearance of polymyxin B

KBIND = THETA(8)	; Binding rate constant of PMB in renal tissues

KUNBIND = THETA(9) ; Unbinding rate constant of PMB in renal tissues

PSELF = THETA(10) ; Permeability rate between Lung and ELF (PAPPLUNG * SALUNG*3.6)

MU_1 = LOG(TVKPREM)

MU_2 = LOG(TVPAPPKID)

MU_3 = LOG(TVSCALAR)

MU_4 = LOG(TVKUSCALAR)

KPREM = EXP(MU_1+ETA(1)) ; Tissue to plasma partition coefficient for remainder compartment

PAPPKID = EXP(MU_2+ETA(2))	; Apparent permeability in kidney

SCALAR = EXP(MU_3+ETA(3)) ; Scalar of tissue to plasma partition coefficient of lung

KUSCALAR = EXP(MU_4+ETA(4)) ; Scalar of unbound tissue to plasma partition coefficient of lung

PSDIFF = PAPPKID*133200*3.6 ; Passive diffusion between renal blood flow and tissues (*PAPPKID* * *SAKID**3.6)

fe = 0.04	; Fraction excreted in urine

FUP = 0.42	; Fraction unbound in plasma

FUELF = 0.546875	;Fraction unbound in ELF

;; ------------ Tissue partition coefficients --------------- ;;

KLUN = 0.14112*SCALAR

KSPL = 0.08694

KLIV = 0.06762

KBRA = 0.06804

KHRT = 0.1344

KSKN = 0.16044

KMUS = 0.04956

KADI = 0.0567

KGI = 0.11844

KULUN = 0.336*KUSCALAR

;; --------Renal and non-renal clearance---------;;

CLH= CLP*(1-fe)

IF(CLH.GE.QLIV) THEN

CLINT=(0.99*QLIV)*QLIV/((QLIV-(0.99*QLIV))*FUP)

CLR = CLP - (CLINT*FUP*QLIV/(CLINT*FUP+QLIV))

ELSE

CLINT = CLH*QLIV/((QLIV-CLH)*FUP)

CLR = CLP*fe

ENDIF

ERH=(FUP*CLINT)/(QLIV+FUP*CLINT)

CLRPMB = FUP*GFR

$DES

CVEN=A(1)/VVEN

CART =A(2)/VART

CBRA = A(3)/VBRA

CHRT = A(4)/VHRT

CMUS = A(5)/VMUS

CSKN = A(6)/VSKN

CADI = A(7)/VADI

CGI = A(8)/VGI

CLUN = A(9)/VLUN

CLIV = A(10)/VLIV

CSPL = A(11)/VSPL

CKIDVAS = A(12)/VKIDVAS

CTUB = A(13)/VTUB

AKIDCEL = A(14)

CKIDCEL = A(14)/VKIDCEL

AKIDDEEP = A(15)

CRES = A(16)/VRES

ADEPSC = A(17)

CELF = A(18)/VELF

;;-----differential equations-------;;

DADT(1) = QLIV*(CLIV/KLIV) + QKID*CKIDVAS + QRES*(CRES/KPREM) + QBRA*(CBRA/KBRA)+ QHRT*(CHRT/KHRT)+ QMUS*(CMUS/KMUS)+ QSKN*(CSKN/KSKN)+ QADI*(CADI/KADI)- QLUN*CVEN + (KASC*ADEPSC)	;Venous

DADT(2) = QLUN*(CLUN/KLUN) - QART*CART	;Artery

DADT(3) = QBRA*CART - QBRA*(CBRA/KBRA) ; Brain

DADT(4) = QHRT*CART - QHRT*(CHRT/KHRT) ; Heart

DADT(5) = QMUS*CART - QMUS*(CMUS/KMUS) ; Muscle

DADT(6) = QSKN*CART - QSKN*(CSKN/KSKN) ; Skin

DADT(7) = QADI*CART - QADI*(CADI/KADI) ; Adipose

DADT(8) = QGI*CART - QGI*(CGI/KGI) ; GI

DADT(9) = QLUN*CVEN - QLUN*(CLUN/KLUN) - PSELF*(CLUN/KULUN) + PSELF*CELF*FUELF	;Lung

DADT(10) = QHEPART*CART + QSPL*(CSPL/KSPL) + QGI*(CGI/KGI) - QLIV*(CLIV/KLIV) - CLINT*FUP*(CLIV/KLIV)	; Liver

DADT(11) = QSPL*CART - QSPL*(CSPL/KSPL) ;Spleen

DADT(12) = QKID*CART - CLRPMB*CKIDVAS - QKID*CKIDVAS - PSDIFF*CKIDVAS + PSDIFF*CKIDCEL ;Kidney vascular

DADT(13) = CLRPMB*CKIDVAS - CLREAB*CTUB - CLR*CTUB	;Tubular

DADT(14) = CLREAB*CTUB - KBIND*AKIDCEL + KUNBIND*AKIDDEEP + PSDIFF*CKIDVAS - PSDIFF*CKIDCEL ;Kidney extravascular

DADT(15) = KBIND*AKIDCEL - KUNBIND*AKIDDEEP ; Kidney binding

DADT(16) = QRES*CART - QRES*(CRES/KPREM) ;Remainder

DADT(17) = -(KASC*ADEPSC) ;SC DEPOT

DADT(18) = PSELF*(CLUN/KULUN) - PSELF*CELF*FUELF	; ELF compartment

$ERROR

CCVEN = A(1)/VVEN

CCLUN = A(9)/VLUN

CCLIV = A(10)/VLIV

CCSPL = A(11)/VSPL

CCKID = (A(12)+A(13)+A(14)+A(15))/VKID

CCELF = A(18)/VELF

DEL=1.0E-30

IF (DVID.EQ.1.) THEN

IPRED=CCVEN

Y=IPRED+EPS(1)+IPRED*EPS(2)

ENDIF

IF (DVID.EQ.2.) THEN

IPRED=CCLUN

Y=IPRED+EPS(1)+IPRED*EPS(2)

ENDIF

IF (DVID.EQ.3.) THEN

IPRED=CCKID

Y=IPRED+EPS(1)+IPRED*EPS(2)

ENDIF

IF (DVID.EQ.4.) THEN

IPRED=CCLIV

Y=CCLIV+EPS(1)+IPRED*EPS(2)

ENDIF

IF (DVID.EQ.5.) THEN

IPRED=CCSPL

Y=IPRED+EPS(1)+IPRED*EPS(2)

ENDIF

IF (DVID.EQ.6.) THEN

IPRED=CCELF

Y=IPRED+EPS(1)+IPRED*EPS(2)

ENDIF

IRES=DV-IPRED

$THETA

(0,0.4)	; 1 TVKPREM

(0,5.9048)	; 2 TVPAPPKID

(0,0.6)	; 3 TVSCALAR

(0,5.9)	; 4 TVKUSCALAR

6.55 FIX	; 5 KASC

0.184 FIX	; 6 CLP

0.0563 FIX	; 7 CLREAB

0.091 FIX	; 8 KBIND

0.0145 FIX	; 9 KUNBIND

1.99 FIX	;10 PSELF

$OMEGA

0	FIX ;1

0	FIX ;2

0	FIX ;3

0	FIX ;4

$SIGMA

0.1	; EPS 1

0.2	;EPS 2

**REFERENCES**

[1]	Davies B, Morris T. Physiological parameters in laboratory animals and humans. Pharm Res 1993;10:1093–5. https://doi.org/10.1023/a:1018943613122.

[2]	Ghofrani HA, Kohstall MG, Weissmann N, Schmehl T, Schermuly RT, Seeger W, et al. Alveolar epithelial barrier functions in ventilated perfused rabbit lungs. Am J Physiol Lung Cell Mol Physiol 2001;280:L896-904. https://doi.org/10.1152/ajplung.2001.280.5.L896.

[3]	Sharma RP, Burgers EJ, Beltman JB. Development of a Physiologically Based Pharmacokinetic Model for Nitrofurantoin in Rabbits, Rats, and Humans. Pharmaceutics 2023;15:2199. https://doi.org/10.3390/pharmaceutics15092199.

[4]	Bussing D, K Shah D. Development of a physiologically-based pharmacokinetic model for ocular disposition of monoclonal antibodies in rabbits. J Pharmacokinet Pharmacodyn 2020;47:597–612. https://doi.org/10.1007/s10928-020-09713-0.

[5]	Viel A, Henri J, Bouchène S, Laroche J, Rolland J-G, Manceau J, et al. A Population WB-PBPK Model of Colistin and its Prodrug CMS in Pigs: Focus on the Renal Distribution and Excretion. Pharm Res 2018;35:92. https://doi.org/10.1007/s11095-018-2379-4.

[6]	Jalil A, Asim MH, Nazir I, Matuszczak B, Bernkop-Schnürch A. Self-emulsifying drug delivery systems containing hydrophobic ion pairs of polymyxin B and agaric acid: A decisive strategy for enhanced antimicrobial activity. Journal of Molecular Liquids 2020;311:113298. https://doi.org/10.1016/j.molliq.2020.113298.

[7]	Dizdarević A, Efiana NA, Phan TNQ, Matuszczak B, Bernkop-Schnürch A. Imine bond formation: A novel concept to incorporate peptide drugs in self-emulsifying drug delivery systems (SEDDS). European Journal of Pharmaceutics and Biopharmaceutics 2019;142:92–100. https://doi.org/10.1016/j.ejpb.2019.06.002.

[8]	Sandri AM, Landersdorfer CB, Jacob J, Boniatti MM, Dalarosa MG, Falci DR, et al. Population Pharmacokinetics of Intravenous Polymyxin B in Critically Ill Patients: Implications for Selection of Dosage Regimens. Clin Infect Dis 2013;57:524–31. https://doi.org/10.1093/cid/cit334.

[9]	Utsey K, Gastonguay MS, Russell S, Freling R, Riggs MM, Elmokadem A. Quantification of the Impact of Partition Coefficient Prediction Methods on Physiologically Based Pharmacokinetic Model Output Using a Standardized Tissue Composition. Drug Metab Dispos 2020;48:903–16. https://doi.org/10.1124/dmd.120.090498.

[10]	Liu X, Chen Y, Yang H, Li J, Yu J, Yu Z, et al. Acute toxicity is a dose-limiting factor for intravenous polymyxin B: A safety and pharmacokinetic study in healthy Chinese subjects. J Infect 2021;82:207–15. https://doi.org/10.1016/j.jinf.2021.01.006.

[11]	Hanafin PO, Kwa A, Zavascki AP, Sandri AM, Scheetz MH, Kubin CJ, et al. A population pharmacokinetic model of polymyxin B based on prospective clinical data to inform dosing in hospitalized patients. Clin Microbiol Infect 2023;29:1174–81. https://doi.org/10.1016/j.cmi.2023.05.018.

[12]	Matcha S, Rao GG, Hanafin PO, Liu X, Yu Z, Zhang J, et al. Population pharmacokinetics of polymyxin B: an individual participant data meta-analysis. Clinical Microbiology and Infection 2026;0. https://doi.org/10.1016/j.cmi.2026.05.036.

[13]	Liu X, Yang L, Wang M, Wang Y, Guo B, Zhang C, et al. Aerosolized delivery resulting in high polymyxin B concentration levels in epithelial lining fluid ensures efficacy in ventilator-associated pneumonia. JAC Antimicrob Resist 2025;7:dlaf023. https://doi.org/10.1093/jacamr/dlaf023.

[14]	Li X, Zhou L, Wang D, Wu Q, Huang X, Zhang H, et al. Population pharmacokinetics study on nebulized and intravenous administration of polymyxin B in patients with pneumonia caused by multidrug-resistant gram-negative bacteria. Antimicrob Agents Chemother 2025;69:e0004425. https://doi.org/10.1128/aac.00044-25.

[15]	Mehta P, Soliman A, Rodriguez-Vera L, Schmidt S, Muniz P, Rodriguez M, et al. Interspecies Brain PBPK Modeling Platform to Predict Passive Transport through the Blood-Brain Barrier and Assess Target Site Disposition. Pharmaceutics 2024;16:226. https://doi.org/10.3390/pharmaceutics16020226.

[16]	Jagdale P, Sepp A, Shah DK. Physiologically-based pharmacokinetic model for pulmonary disposition of protein therapeutics in humans. J Pharmacokinet Pharmacodyn 2022;49:607–24. https://doi.org/10.1007/s10928-022-09824-w.

[17]	Gaohua L, Wedagedera J, Small B, Almond L, Romero K, Hermann D, et al. Development of a Multicompartment Permeability-Limited Lung PBPK Model and Its Application in Predicting Pulmonary Pharmacokinetics of Antituberculosis Drugs. CPT: Pharmacometrics & Systems Pharmacology 2015;4:605–13. https://doi.org/10.1002/psp4.12034.

[18]	Appendix F: Estimating Human Equivalent Concentrations Using the U.S. EPA Default Approach 2008.

[19]	Huang W, Isoherranen N. Novel Mechanistic PBPK Model to Predict Renal Clearance in Varying Stages of CKD by Incorporating Tubular Adaptation and Dynamic Passive Reabsorption. CPT: Pharmacometrics & Systems Pharmacology 2020;9:571–83. https://doi.org/10.1002/psp4.12553.

[20]	Covelli J, Ruszaj D, Straubinger R, Li J, Rao GG. The development and validation of a simple liquid chromatography-tandem mass spectrometry method for polymyxin B1 and B2 quantification in different matrices. J Chromatogr B Analyt Technol Biomed Life Sci 2017;1065–1066:112–8. https://doi.org/10.1016/j.jchromb.2017.09.031.

[21]	Rennard SI, Basset G, Lecossier D, O’Donnell KM, Pinkston P, Martin PG, et al. Estimation of volume of epithelial lining fluid recovered by lavage using urea as marker of dilution. J Appl Physiol (1985) 1986;60:532–8. https://doi.org/10.1152/jappl.1986.60.2.532.

[22]	Wen H, Sadiq MW, Friberg LE, Svensson EM. Translational Physiologically Based Pharmacokinetic Modeling to Predict Human Pulmonary Kinetics After Lung Delivery. CPT Pharmacometrics Syst Pharmacol 2025;14:796–806. https://doi.org/10.1002/psp4.13316.

[23]	Stephens RH, Benjamin AR, Walters DV. Volume and protein concentration of epithelial lining liquid in perfused in situ postnatal sheep lungs. J Appl Physiol (1985) 1996;80:1911–20. https://doi.org/10.1152/jappl.1996.80.6.1911.

[24]	Mahadevan R, Garcia E, Sharma R, Qiu H, Elsheikh A, Parambi R, et al. A mechanism-based pharmacokinetic/pharmacodynamic analysis of polymyxin B-based combination therapy against carbapenem-resistant Klebsiella pneumoniae isolates with diverse phenotypic and genotypic resistance mechanisms. Antimicrob Agents Chemother 2025:e0078225. https://doi.org/10.1128/aac.00782-25.

[25]	Liu J, Shao M, Xu Q, Liu F, Pan X, Wu J, et al. Low-dose intravenous plus inhaled versus intravenous polymyxin B for the treatment of extensive drug-resistant Gram-negative ventilator-associated pneumonia in the critical illnesses: a multi-center matched case-control study. Ann Intensive Care 2022;12:72. https://doi.org/10.1186/s13613-022-01033-5.

[26]	Bulitta JB, Landersdorfer CB. Performance and Robustness of the Monte Carlo Importance Sampling Algorithm Using Parallelized S-ADAPT for Basic and Complex Mechanistic Models. AAPS J 2011;13:212–26. https://doi.org/10.1208/s12248-011-9258-9.

[27]	Bulitta JB, Bingölbali A, Shin BS, Landersdorfer CB. Development of a New Pre- and Post-Processing Tool (SADAPT-TRAN) for Nonlinear Mixed-Effects Modeling in S-ADAPT. AAPS J 2011;13:201–11. https://doi.org/10.1208/s12248-011-9257-x.
